# Bioengineered constructs combined with exercise enhance stem cell-mediated treatment of volumetric muscle loss

**DOI:** 10.1038/ncomms15613

**Published:** 2017-06-20

**Authors:** Marco Quarta, Melinda Cromie, Robert Chacon, Justin Blonigan, Victor Garcia, Igor Akimenko, Mark Hamer, Patrick Paine, Merel Stok, Joseph B. Shrager, Thomas A. Rando

**Affiliations:** 1 Department of Neurology and Neurological Sciences, Stanford University School of Medicine, Stanford, California 94305, USA; 2 Paul F. Glenn Laboratories for the Biology of Aging, Stanford University School of Medicine, Stanford, California 94305, USA; 3 Center for Tissue Regeneration, Restoration and Repair, Veterans Affairs Hospital Palo Alto, California 94036, USA; 4 Erasmus Medical Center, Department of Hematology and Department of Pediatrics, Rotterdam 3000, The Netherlands; 5 Division of Thoracic Surgery, Department of Cardiothoracic Surgery, Stanford University School of Medicine and VA Palo Alto Health Care System, Stanford, California 94305, USA

## Abstract

Volumetric muscle loss (VML) is associated with loss of skeletal muscle function, and current treatments show limited efficacy. Here we show that bioconstructs suffused with genetically-labelled muscle stem cells (MuSCs) and other muscle resident cells (MRCs) are effective to treat VML injuries in mice. Imaging of bioconstructs implanted in damaged muscles indicates MuSCs survival and growth, and *ex vivo* analyses show force restoration of treated muscles. Histological analysis highlights myofibre formation, neovascularisation, but insufficient innervation. Both innervation and *in vivo* force production are enhanced when implantation of bioconstructs is followed by an exercise regimen. Significant improvements are also observed when bioconstructs are used to treat chronic VML injury models. Finally, we demonstrate that bioconstructs made with human MuSCs and MRCs can generate functional muscle tissue in our VML model. These data suggest that stem cell-based therapies aimed to engineer tissue *in vivo* may be effective to treat acute and chronic VML.

## Abstract (web summary)

Volumetric muscle loss leads to functional muscle impairment, and current stem cell-based treatments show limited efficacy. Here, the authors generate a stem cell scaffold, implant it in mice, and show that an exercise regimen enhances innervation and restoration of muscle function in mice.

Volumetric muscle loss (VML) is defined as an irrecoverable injury sustained by skeletal muscle and characterized by a permanent loss of tissue structure and function^1^. The result is life-long disability attributed to scar tissue formation and structural changes^2^,^3^. Currently, state-of-the-art treatments are limited to scar tissue debridement and placement of muscle flaps around the site of tissue defects^4^,^5^. Unfortunately, these two clinical options are inefficient and the outcomes remain aesthetically and functionally deficient^2^,^6^.

At the root of muscle injury repair and regeneration, exists a population of resident adult muscle stem cells (MuSCs), or ‘satellite cells’, that, in uninjured muscle, reside embedded under the basal laminae of muscle fibres in a quiescent state^7^,^8^. In response to even minor injuries, MuSCs activate and give rise to myogenic progenitor cells that can fully restore the original structure and function of the tissue^3^,^9^. Moreover, during regenerative processes of the muscle tissue, MuSCs are supported by other cell types^10^,^11^. However, despite their remarkable regenerative potential, MuSCs fail to restore tissue structure and function when faced with the extensive damage associated with VML lesions^10^,^12^. Importantly, recent findings showed that acellular muscle architecture is indeed required to properly guide MuSCs during muscle regeneration^13^.

It has been suggested that transplantation of scaffolds composed of extracellular matrix (ECM) proteins promote the repair of VML injury by providing a structural and biochemical framework^14^,^15^. Recent reports indicate that culturing myogenic cells in decellularized ECM-based scaffolds prior to transplantation results in some functional and structural repair of VML lesions^16^,^17^,^18^. Other studies using hydrogels to support transplanted cells have demonstrated that MuSCs can partially restore the structure and function of injured muscle^17^,^19^,^20^. Nevertheless, a tissue engineering system capable of restoring even near normal mature muscle, vasculature and ECM composition to the damaged tissue has yet to be achieved. More importantly, achieving *de novo* innervation of regenerated myofibres remains a critical step for functional restoration of VML injuries.

Here, we engineer a novel VML treatment based on a strategy designed to mimic the endogenous niche of MuSCs within transplantable bioconstructs. We find that bioconstructs generated with MuSCs and muscle resident cells (MRCs) are capable of restoring structure and function of muscles with acute and chronic VML injuries. A regimen of exercise further improves innervation and force production *in vivo*. Finally, bioconstructs generated with human MuSCs and MRCs result in functional muscle tissue in our VML model. These advances in stem cell-based methods may offer new tools to design treatments for VML in the clinical setting.

## Results

### Characterization of the VML model

**Figure 1:**
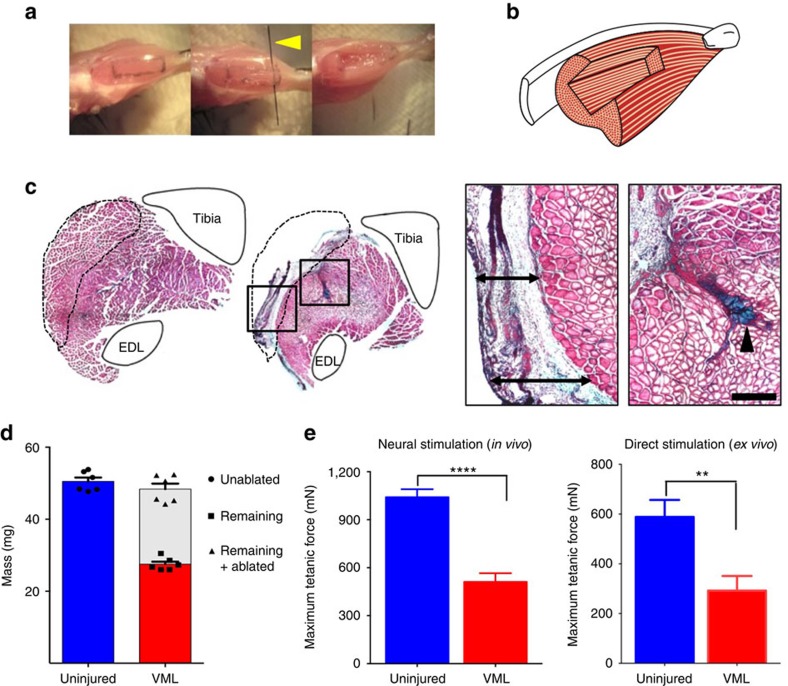
Mouse model of VML results in irrecoverable loss of function and structure. (**a**) VML surgical procedure. From left to right, a photographic sequence shows tattooing and surgical ablation with a micro-blade (indicated by a yellow arrowhead) of a defined mass from a TA muscle. (**b**) Model of TA muscle with ablation injury. A rendition of the rectangular pocket created within the TA by the surgical excision is shown. (**c**) Representative H&E staining of whole TA muscle cross-sections. An uninjured TA muscle (left panel) and a TA muscle 30 days following VML injury (right panel) are shown. The tibia and the extensor digitorum longus (EDL) muscle have been drawn for each image to facilitate the orientation and the anatomy of the TA muscles. A dashed line indicates the area corresponding to the muscle area removed in the surgical procedure. For the VML model image, the boxed areas are shown in higher magnification to the right (scale bar=2 mm). Left box: peripheral fibrotic scarring is observed in place of the excised muscle (indicated by arrow bars). Right box: a fibrotic scar can be seen extending into the belly of the TA muscle (indicated by arrow head) (scale bar=500 μm). (**d**) Quantification of TA muscle tissue masses. TA muscles were weighed immediately following dissection. For muscles subjected to VML injuries, the ablated muscle tissues were immediately weighed (white bar) and added to the muscle mass remaining 30 days after the ablation (red bar). Comparisons were made to unablated muscles. (**e**) Force production, measured through a force transducer, of TA muscles 30 days after VML injuries compared with uninjured control TA muscles. Contractions were induced *in vivo* through direct sciatic nerve stimulation (left graph) or *ex vivo* by inducing contraction directly through electrical stimulation in a culture bath (right graph) (*n*=6). Data are±s.e.m. For statistical analysis, *t*-tests were used. *P<0.05; **P<0.001; ****P<0.00001.

With the goal of developing treatment strategies capable of ameliorating the regenerative deficits of VML injuries, we first sought to generate a dependable, reproducible and anatomically accessible murine VML model. Based on previous models^21^,^22^,^23^, we developed a VML model using the tibialis anterior (TA) muscle of mice. We used a 40% surgical ablation of the TA muscle and we assessed the injured muscles over the next 30 days (Fig. 1a,b). As expected, we observed that the ablated TA muscle was unable to regenerate to any meaningful extent (Fig. 1c). To further characterize this lasting deficit, we weighed the ablated muscles and found a permanent loss of muscle mass compared with uninjured muscles (Fig. 1d). By adding the mass of each ablated mass with the mass of the remaining tissue 30 days after injury and comparing it with that of the uninjured contralateral TA muscle, we confirmed that no significant compensatory hypertrophy or partial recovery had occurred (Fig. 1d). *In vivo* muscle force production of the TA muscles was quantified by stimulating the sciatic nerve and directly measuring biomechanics *in situ* by attaching the transducer to the TA distal tendon. The ablated TA muscles showed ∼40% reduction in force when compared with uninjured muscles (Fig. 1e). Similarly, direct *ex vivo* electrical stimulation of the dissected muscles resulted in force reduction of ∼50% compared with uninjured muscles (Fig. 1e). These results suggest that our defined surgical ablation resulted in consistent and permanent loss of TA mass and function, characteristic of VML injuries.

### Scaffolds with MuSCs result in *de novo* myofibres *in vivo*

Previous studies in which MuSCs were directly introduced into damaged muscle have resulted in limited *de novo* muscle formation^9^,^24^. Many investigators have achieved better stem or progenitor cell engraftment by first implanting the cells onto 3D scaffolds prior to transplantation^19^,^20^,^25^. Thus, we sought to test whether MuSCs, transplanted in combination with a 3D scaffold composed of decellularized muscle tissue, might display enhanced regenerative capacity compared with decellularized muscle alone or MuSCs alone.

To assess the ability of MuSCs to generate new muscle fibres in our VML model, we isolated MuSCs from the hind limb muscles of mice using fluorescent activated cell sorting (FACS). We injected these MuSCs into decellularized TA muscle scaffolds in preparation for transplantation (Supplementary Fig. 1a). However, direct injection of MuSCs in suspension into the decellularized scaffold proved to be an ineffective means to prepare cells for transplantation because of poor cellular retention within the scaffold. We thus tested whether suspending cells in more viscous solutions prior to injection into the scaffolds would enhance the retention of cells.

Hydrogels have been used extensively as biocompatible substrates for maintaining viable 3D MuSC cultures in both *in vitro* and *in vivo* applications^19^,^26^. We screened different hydrogel formulations for their capacity to sustain MuSC viability. We isolated MuSCs from mice expressing the bioluminescent reporter proteins Luciferase and GFP (FVB-Tg(CAG-luc,-GFP)L2G85Chco/J) and cultured them in various experimental hydrogel formulations (see Supplementary Table 1). After 48 h in culture, we administered luciferin and measured bioluminescence using an imaging system (IVIS) (Supplementary Fig. 1b). Cells cultured in a Collagen I hydrogel or in an ECM hydrogel emitted the highest bioluminescent signals, suggesting that they were the most effective in maintaining cell viability.

Upon demonstrating the highest propensity of MuSCs to survive in these two hydrogels *in vitro*, we next assessed their contribution to our cellular reconstitution and transplantation strategy. First, we suffused MuSCs in solutions of ECM or Collagen I and microinjected them into the scaffolds. Completed bioconstructs were deemed ready for transplantation once the gels had formed and cured (typically 30–60 min). We then transplanted bioconstructs generated into our VML model and analysed the muscles histologically after 10 days. Within the bioconstructs, the myofibres stained positive for embryonic Myosin Heavy Chain (eMHC) (Supplementary Fig. 1c), providing evidence of *de novo* myofibre formation. Bioconstructs generated using MuSCs suspended in ECM-based hydrogel (compared with Collagen I hydrogel or no hydrogel) resulted in the highest number of myofibres per region of interest (Supplementary Fig. 1d).

**Figure 2:**
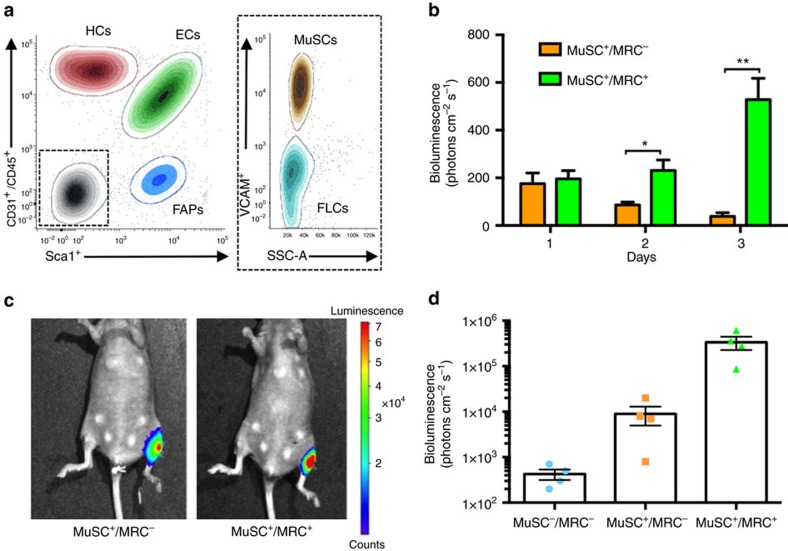
MRCs support MuSCs in *de novo* myofibre formation. (**a**) Representative FACS plot of MRCs. Lower limb muscles were dissected and digested to obtain a mononucleated cellular suspension. These cells were marked using cell specific surface antigens as described in the ‘Results’ and in the ‘Methods’ sections and were analysed using FACS. Five populations were isolated: muscle stem cells (MuSCs), hematopoietic cells (HCs), endothelial cells (ECs), fibro-adipogenic progenitor cells (FAPs) and fibroblast-like cells (FLCs). The relative percentages of each cell population are 10%, 25%, 39%, 8% and 18%, respectively (*n*=6). (**b**) Quantified results of *in vitro* bioluminescence generated from cultured bioconstructs containing Luc^+^ MuSCs, either alone (MuSC^+^/MRC^−^) or in combination with Luc^−^ MRCs (MuSC^+^/MRC^+^). Bioconstructs were cultured for three days, and bioluminescence was measured each day (*n*=4). (**c**) Representative images of bioluminescence measured from mice 10 days after transplantation of bioconstructs in left TA muscles immediately following VML injury (**d**) Quantified results of non-invasive imaging of transplanted bioconstructs. Bioconstructs with no cells (MuSC^−^/MRC^−^), Luc^+^ MuSCs (MuSC^+^/MRC^−^), or Luc^+^ MuSCs in addition to Luc^−^ MRCs (MuSC^+^/MRC^+^) were transplanted into TA muscles that had received VML injuries. Bioluminescence was measured 10 days following transplantation (*n*=4). Data are±s.e.m. For statistical analysis, *t*-tests were used. *P<0.05; **P<0.001.

Muscle tissue formation by MuSCs is supported by a number of other MRCs^25^,^27^,^28^. These MRCs include fibro-adipogenic progenitors (FAPs), macrophages and endothelial cells^11^,^29^,^30^,^31^,^32^. We used FACS to isolate four distinct populations of mononucleated cells based on cell surface markers^33^,^34^: (1) MuSCs (VCAM^+^/CD45^−^/CD31^−^/Sca1^−^); (2) hematopoietic cells, or HCs (VCAM^−^/CD45^+^/CD31^−^/Sca1^−^); (3) endothelial cells, or ECs (VCAM^−^/CD45^−^/CD31^+^/Sca1^+^); (4) FAPs (VCAM^−^/CD45^−^/CD31^−^/Sca1^+^); and (5) a population which was negative for all cell surface proteins (VCAM^−^/CD45^−^/CD31^−^/Sca1^−^) (Fig. 2a). *In vitro* characterization of this marker-negative population revealed it to be enriched in fibroblast-like cells (FLCs) (Supplementary Fig. 2a,b).

We assessed how supplementing our bioconstructs with these various cell types (which, together, we refer to as MRCs) might enhance the efficacy of MuSCs in mediating *de novo* myofibre formation following transplantation into VML lesions. We generated MuSC^+^/MRC^−^ and MuSC^+^/MRC^+^ bioconstructs containing Luciferase-expressing (Luc^+^) MuSCs with MRCs isolated from Luc^−^ mice. We reconstituted scaffolds with ECM-based hydrogels suffused either with 150,000 MuSCs alone (MuSC^+^/MRC^−^) or with 150,000 MuSCs plus 350,000 MRCs (MuSC^+^/MRC^+^). For the MuSC^+^/MRC^+^ formulations, the relative numbers of cells from each population were based upon the cell ratios recorded during the initial FACS analysis (Fig. 2a and Methods). We cultured them for up to 3 days, comparing MuSC survival by measuring bioluminescence over time. Much higher bioluminescence signals, indicative of much higher MuSC survival, were observed in bioconstructs into which both MuSCs and MRCs had been introduced (Fig. 2b).

We then tested the myogenic potential of our bioconstructs *in vivo*. We again generated bioconstructs containing either MuSC^+^/MRC^−^ or MuSC^+^/MRC^+^ populations, or bioconstructs containing no cells at all (MuSC^−^/MRC^−^ formulation; hydrogel alone). All MuSCs expressed Luciferase. We then transplanted these bioconstructs into the TA muscles of immunocompromised (NOD.CB17-*Prkdcscid*/J or NOD-SCID) mice with acute VML injuries (Supplementary Fig. 3), and we measured bioluminescence non-invasively 30 days post transplantation. We found that MuSC^+^/MRC^+^ bioconstructs emitted bioluminescence signals almost 40 times higher than those of MuSC^+^/MRC^−^ bioconstructs (Fig. 2c,d). Together, these results suggest that MRCs support MuSC viability within bioconstructs *in vitro* and *in vivo*.

### ECs are necessary and sufficient to sustain MuSCs

Recreating and supporting the extended vascular networks required to sustain growth of new engineered tissue during regenerative interventions remains a critical challenge^25^,^35^. ECs participate during the regenerative process of injured muscles by providing trophic support to MuSCs and contributing in the angiogenic process^32^,^36^,^37^,^38^. We thus asked whether ECs might be having a critically specific role in sustaining MuSCs *in vivo,* within the transplanted bioconstructs. To test this, we generated bioconstructs with Luc^+^ MuSCs alone or along with ECs (unlabelled) and transplanted them into VML injuries. When we measured bioluminescence 21 days later, we found that ECs partially rescued the loss of MuSC viability measured when transplanted into bioconstructs without MRCs (Supplementary Fig. 4a). In addition, when we transplanted bioconstructs containing Luc^+^ MuSCs and either Luc^−^ MRCs including ECs or Luc^−^ MRCs depleted of ECs, we found that supplementing MuSCs with MRC population that did not contain ECs was less effective than with the population of MRCs containing ECs (Supplementary Fig. 4a). These results suggest that ECs are required for the enhancing effect of the total MRC population to sustain MuSCs viability, expansion and engraftment within bioconstructs transplanted in VML injuries, and also that ECs alone are sufficient to produce nearly the full effect of the total MRC population.

We next asked what the fate of transplanted ECs was during the process of *de novo* tissue formation. We generated bioconstructs containing either Luc^+^ MuSCs and Luc^−^ MRCs or Luc^−^ MuSCs and Luc^+^ ECs together with the other Luc^−^MRCs. We then monitored bioluminescence from transplanted MuSCs or ECs non-invasively. We found that both MuSCs and ECs survived and expanded within the bioconstructs up to 21 days after the transplantation (Supplementary Fig. 4b,c). These results indicate that ECs can grow within transplanted bioconstructs, persisting in the *de novo* tissue.

Finally, to test whether donor-derived ECs were not only providing trophic support to MuSCs, but also engrafting and possibly participating in *de novo* vasculogenesis, we transplanted bioconstructs containing DS-Red^−^ MuSCs and DS-Red^+^ ECs together with the rest of DS-Red^−^ MRCs. Surprisingly, immunohistological analysis of the transplanted TA muscles 30 days later showed extensive distribution of donor-derived ECs in vascular structures around myofibres within transplanted bioconstructs (Supplementary Fig. 4d). These results suggest that ECs are a critical MRC population capable of engrafting and contributing to vasculogenesis within *in vivo* engineered *de novo* muscle tissue.

### Perfusion in a bioreactor enhances MuSCs viability

**Figure 3:**
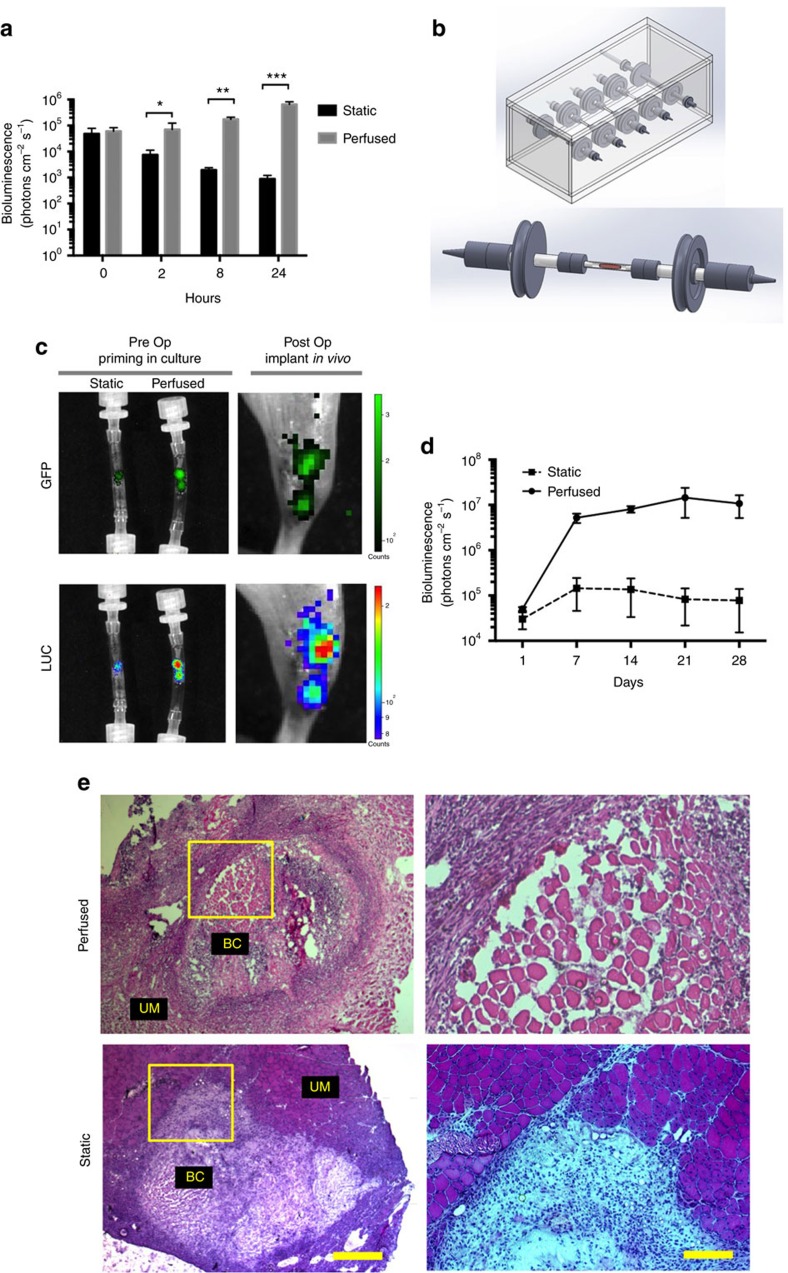
Perfusion of bioconstructs in a bioreactor improves MuSC efficacy. (**a**) Perfusion of bioconstructs sustains MuSC viability *in vitro*. Bioconstructs were reconstituted with Luc^+^ MuSCs and Luc^−^ MRCs and then cultured for 24 h. Bioluminescence was measured at different time points, as indicated (*n*=6). (**b**) Model of the bioreactor designed to perfuse cultured bioconstructs. An autoclavable plexiglass chamber, capable of holding, in parallel, five independent tubing lines, is shown (top panel). These lines were connected to an external pump. Each line contained one tubular culture chamber capable of hosting one bioconstruct (bottom panel). (**c**) Representative bioluminescence and fluorescence images of bioconstructs before and after transplantation. Bioconstructs were generated with Luc^+^/GFP^+^ MuSCs and Luc^−^/GFP^−^ MRCs, cultured under either perfused or static conditions, and imaged non-invasively before transplantation (Pre Op, left panels). Bioconstructs that had been imaged previously *in vitro* were imaged non-invasively *in vivo* immediately following transplantation (Post Op, right panels). (**d**) Quantitative results comparing bioluminescence of perfused and static bioconstructs at periodic intervals following transplantation into VML injuries (*n*=4). (**e**) (Left panels) Representative H&E-stained cross-sections of muscles 10 days after receiving perfused or static bioconstruct transplantation to treat VML injuries. Bioconstructs (BC) are shown adjacent to unablated muscle (UM) (scale bar=500 μm). (Right panels) The regions outlined by the yellow boxes in the left panel are magnified here (scale bar=200 μm Data are±s.e.m. For statistical analysis, *t*-tests were used. *P<0.05; **P<0.001; ***P<0.0001.

Culturing cells within 3D substrates results in a loss of cell viability^9^,^39^. During the process of generating bioconstructs with Luc^+^ MuSCs, we observed substantial bioluminescent signal decay *in vitro* within two hours of reconstitution, continuing up to 24 h (Fig. 3a). Perfusion systems have proven a practical tool for remedying the loss of cell viability in 3D culture conditions^35^,^39^,^40^. We thus sought to establish a perfusing bioreactor to be used immediately after curing of the hydrogel to test whether this would enhance MuSC viability.

To create perfusing bioreactors, we generated tubular culture chambers made of Tygon E-3603 biopolymer with an inner diameter similar to the scaffolds. Inside these chambers, bioconstructs were fabricated by reconstituting scaffolds using a micromanipulator to control the coordinates of injection and a micropump to regulate the flow rate and volume of the hydrogel-cell suspension being injected. Finally the culture chambers were connected to a bioreactor allowing perfusion of the bioconstructs with recirculating media (Fig. 3b). We initially validated perfusion performance in cell-free bioconstructs by introducing a blue dye into the recirculating media. We found that the dye diffused efficiently throughout the bioconstructs (Supplementary Fig. 5a). We then tested the effects of perfusion of culture medium on the survival of cells within the bioconstructs by measuring the bioluminescence of bioconstructs reconstituted with Luc^+^ MuSCs plus MRCs. Over the next 24 h, the bioluminescent signal in bioconstructs cultured under static conditions decreased significantly relative to initial measurements. Conversely, when we perfused the bioconstructs with culture medium, the bioluminescence increased over time (Fig. 3a).

To test whether these perfused bioconstructs resulted in improved MuSC engraftment and *de novo* muscle fibre formation *in vivo*, we generated MuSC^+^/MRC^+^ bioconstructs using MuSCs that had been transduced with a lentivirus expressing Luciferase and GFP. After culturing these bioconstructs under either static or perfused conditions for 24 h, we transplanted them into TA muscles of mice with acute VML injuries. We imaged bioluminescence of these bioconstructs immediately prior to transplantation and then again immediately after transplantation to establish the initial readings (Fig. 3c). We then monitored and recorded bioluminescent signalling non-invasively over the next 30 days (Fig. 3d). Whereas the bioluminescent signals from TA muscles transplanted with static bioconstructs increased several fold over that time, the signals from muscles transplanted with the perfused bioconstructs increased by almost three orders of magnitude (Fig. 3d). After 30 days, muscles transplanted with perfused bioconstructs emitted bioluminescent signals more than two orders of magnitude greater than those transplanted with static bioconstructs.

Consistent with bioluminescence imaging, immunohistochemical analysis of bioconstructs 10 days following transplantation revealed regions of Luciferase^+^/GFP^+^ myofibres in muscles transplanted with the perfused bioconstructs (Supplementary Fig. 5b); only rare Luc^+^/GFP^+^ myofibres were observed in muscles transplanted with the static bioconstructs. Histological analysis confirmed the presence of muscle fibres within the transplanted perfused bioconstructs (Fig. 3e). Only rarely were myofibres found within transplanted static bioconstructs. These results suggest that treatment of VML lesions using MuSCs incorporated into a scaffold can be enhanced by perfusing the cell/hydrogel/scaffold bioconstructs to maintain cell viability prior to transplantation.

### Bioconstructs restore active force generation in VML

**Figure 4:**
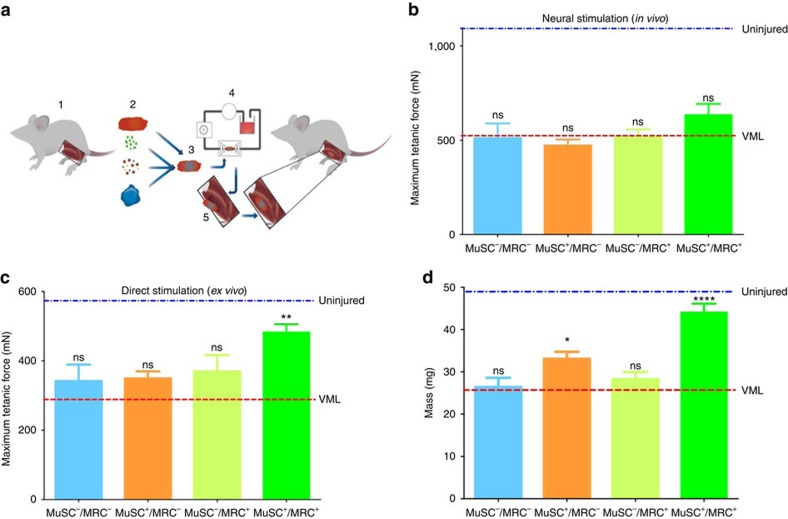
Treatment of acute VML restores mass and force production. (**a**) Schematic model of our VML treatment procedure using bioconstructs. TA muscles are isolated from donor mice (1). These muscles are used to obtain either: decellularized scaffolds, MuSCs, MRCs or ECM proteins to generate hydrogels (2). Scaffolds are reconstituted with isolated cells and hydrogel to generate bioconstructs (3). Bioconstructs are cultured in a bioreactor while the hydrogel cured, allowing media to perfuse across the bioconstructs (4). Once ready, bioconstructs are transplanted into VML injured TA muscles (5). (**b**) *In vivo* force production measurements of TA muscles treated with different bioconstructs following VML injury. After 30 days, the distal tendons were attached to a force transducer and contractions were induced through sciatic nerve stimulation (*n*=8). (**c**) *Ex vivo* force production measurements. The same muscles measured in **b** were then dissected and cultured in a chamber. The distal tendons were attached to a transducer and contractions were induced electrically in the culture bath (*n*=8). (**d**) The mass of each TA muscle was measured following *ex vivo* force measurements (*n*=6). In (**b**–**d**) average values of muscles that did not received VML injuries are labelled ‘uninjured’ and are indicated by a blue dotted line; average values of muscles that received a VML injury without any treatment are labelled ‘VML’ and are indicated by a red dotted line. Data are±s.e.m. For statistical analysis, *t*-tests were used. **P<0.001; ****P<0.00001, n.s.: not significant.

The development of a successful stem cell-based strategy for repairing VML must restore not only muscle structure but also muscle function. To test whether we could repair the loss of function measured earlier in our force production experiments (Fig. 1e,f), we generated MuSC^+^/MRC^+^, MuSC^+^/MRC^−^, MuSC^−^/MRC^+^ and MuSC^−^/MRC^−^ bioconstructs to treat acute VML lesions using all of the optimisations described above (Fig. 4a). The MuSCs used were isolated from mice expressing the reporter fluorescence protein ‘enhanced YFP’ (eYFP; isolated from a Pax7Cre^ER^/ROSA26^eYFP^ mouse)^41^. We then surgically transplanted these bioconstructs into TA muscles with acute VML injuries. After 30 days, we measured isometric tetanic forces of these muscles. First, we measured the force generated *in vivo* by connecting the distal TA tendon to a force transducer and electrically stimulating the sciatic nerve. None of the treatments showed a significant improvement in force production when compared with untreated VML conditions using this form of stimulation (Fig. 4b). As an alternative, we measured muscle forces *ex vivo*. When directly stimulated in this manner, the muscles transplanted with MuSC^+^/MRC^+^ bioconstructs generated active forces that were significantly greater than untreated VML muscles (Fig. 4c), suggesting that the force measurements by sciatic nerve stimulation were limited by the innervation of new myofibres as opposed to their intrinsic force-generating potential. By contrast, MuSC^+^/MRC^−^, MuSC^−^/MRC^+^ and MuSC^−^/MRC^−^ bioconstructs did not show any significant improvement compared with untreated VML conditions (Fig. 4c). Furthermore, TA muscle mass was partially restored in the MuSC^+^/MRC^+^ treatment group and, to a lesser extent, in the MuSC^+^/MRC^−^ treatment group (Fig. 4d). In the MuSC^−^/MRC^+^ and MuSC^−^/MRC^−^ controls, the increase in mass was essentially that of the transplanted bioconstructs. These results suggest that MuSC^+^/MRC^+^ bioconstructs are capable of functionally restoring muscle tissue by generating *de novo* myofibres, but may not be functionally innervated 30 days after treatment.

### Bioconstructs restore muscle and vascular structure

**Figure 5:**
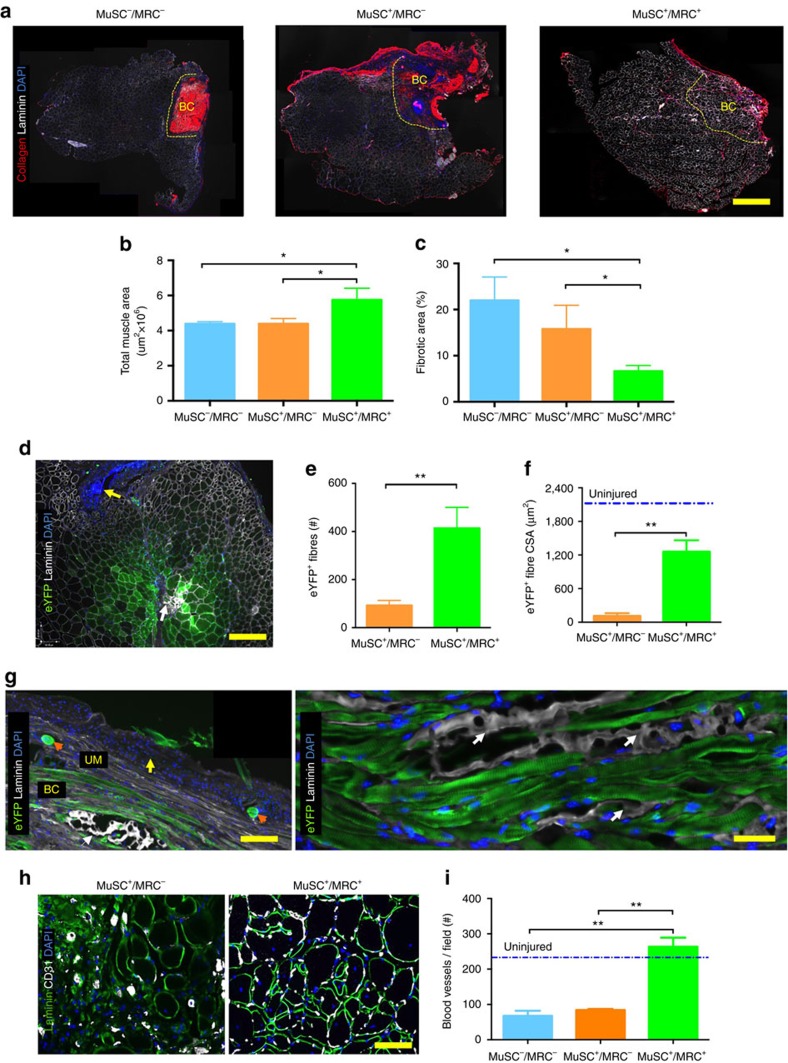
Treatment of VML improves and restores tissue structure. (**a**) Representative immunofluorescence (IF) images of cross-sections of VML-injured TA muscles treated with different bioconstructs. Three of the same muscles used for force productions in Fig. 4 were sectioned and immunostained (scale bar=1 mm). (**b**) Measurements of whole muscle tissue areas for all muscles assessed in Fig. 5 (*n*=6). (**c**) Measurements of fibrotic tissue areas within total muscle areas quantified in **b** (*n*=6). (**d**) Representative IF cross-sectional images showing donor-derived eYFP^+^ myofibres following treatment. Muscles treated with bioconstructs generated using eYFP^+^ MuSCs and eYFP^−^ MRCs were harvested after taking force measurements. The panel shows a region in which a bioconstruct was implanted within a VML-injured TA muscle. The yellow arrow indicates peripheral fibrotic scarring as illustrated in Fig. 1. The white arrow indicates residual scaffold surrounded by *de novo* myofibres (scale bar=500 μm). (**e**) Quantification of the number of eYFP^+^ myofibres in muscles treated with bioconstructs generated with either eYFP^+^ MuSCs alone or together with eYFP^−^ MRCs (*n*=6). (**f**) Quantification of cross-sectional areas of the myofibres counted in **e** (*n*=6). (**g**) Representative IF longitudinal images showing donor-derived eYFP^+^ myofibres in transplanted bioconstructs (scale bar=500 μm). The left panel shows a low magnification image of a TA muscle centred on the transplanted bioconstruct region (scale bar=500 μm). The yellow arrow indicates the peripheral fibrotic scarring adjacent to the unablated muscle (UM), which is overlying *de novo* muscle fibres within the bioconstruct (BC). The orange arrows indicate two stiches securing the BC within the tissue. The white arrow indicates a region of residual scaffold. The right panel shows at higher magnification image of donor-derived eYFP^+^ myofibres. The white arrows again indicate regions of residual scaffold (scale bar=50 μm). (**h**) Representative IF images showing blood vessels structures formed by endothelial cells (CD31^+^) within regions of transplanted bioconstructs (scale bar=100 μm). (**i**) Quantification of blood vessels from regions of transplanted bioconstructs in muscles with VML injuries (*n*=6). Data are±s.e.m. For statistical analysis, *t*-tests were used. **P<0.001; ***P<0.0001.

To test whether the observed restoration of biomechanical force production was associated with structural repair of the VML injury, we performed histological analysis on the TAs from the experiments described above following force measurements. Morphometric analysis of whole TA cross-sections revealed that, relative to untreated VML-injured muscles, only muscles treated with MuSC^+^/MRC^+^ bioconstructs showed a statistically significant increase in size as measured by cross-sectional areas (CSAs) (Fig. 5a,b). Bioconstructs reconstituted with the MuSC^+^/MRC^+^ populations also exhibited markedly reduced fibrosis (Fig. 5a,c). From these data, we concluded that the current bioconstruct reconstitution represents a treatment strategy that provides a pro-regenerative environment in regions that are severely impaired by VML injuries.

Our next experiment was designed to further characterize the contribution of donor-derived cells to the formation of new muscle fibres. Therefore, we transplanted bioconstructs containing donor MuSCs expressing eYFP to quantify *de novo* eYFP^+^ myofibres within those regions. We found at 10 days following transplantation that treatment with MuSC^+^/MRC^+^ bioconstructs generated donor-derived myofibres adjacent to scaffold (Supplementary Fig. 6a). Analysis of cross-sections and longitudinal-sections of muscles 30 days following bioconstructs transplantation revealed donor-derived myofibres homogenously distributed throughout the injured regions (Fig. 5d,g). The degree of maturation of these myofibres was reflected by sarcomeric striations (Supplementary Fig. 6b). MuSC^+^/MRC^+^ bioconstructs yielded greater numbers of eYFP^+^ myofibres (413±87) than did the MuSC^+^/MRC^−^ (92±21) or MuSC^−^/MRC bioconstructs (no fibres) (Fig. 5e). Furthermore, eYFP^+^ myofibres in muscles transplanted with MuSC^+^/MRC^+^ bioconstructs had greater CSAs than did those in muscles transplanted with MuSC^+^/MRC^−^ bioconstructs (Fig. 5f). Together, these results provide further evidence that the generation of new muscle tissue in VML injuries transplanted with bioconstructs containing MuSCs is enhanced by the inclusion of MRCs.

Immunohistochemical staining for blood vessels, defined as CD31^+^ structures, revealed that the MuSC^+^/MRC^+^-treated muscles were more extensively vascularized compared with muscles treated with MuSC^+^/MRC^−^ or MuSC^−^/MRC^−^ bioconstructs (Fig. 5h,i). To confirm that the CD31^+^ structures were functionally engrafted with the host vasculature, we injected Isolectin intravenously into the circulation and fixed the muscles 10 min following the injection. Immunohistochemistry confirmed the presence of CD31^+^/Isolectin^+^ vessels within the transplanted regions, surrounding donor-derived fibres (Supplementary Fig. 7a,b). Our findings, consistent with our results on the role of ECs (Supplementary Fig. 4), suggest that treatments containing MRCs help to overcome the challenge of vascularisation by supporting *de novo* functional vasculogenesis in concert with the *de novo* muscle formation.

### Exercise improves innervation and functional recovery

Next, we turned our attention to the discrepancy observed between forces measured using direct muscle stimulation compared with sciatic nerve stimulation (Fig. 4c versus Fig. 4b). We hypothesized that this discrepancy might be due to the fact that new muscle tissues generated by the transplanted bioconstructs had not been efficiently innervated in our acute VML model. To test this hypothesis, we analysed the neuromuscular junctions (NMJs) associated with regenerated myofibres within our transplanted bioconstructs using fluorescently-labelled α-bungarotoxin (αBTX). We found fewer mature NMJs 30 days after treatment in regions of the transplanted bioconstructs in both the MuSC^+^/MRC^+^ and the MuSC^+^/MRC^−^ treatment groups compared with unablated regions of the same muscles (Supplementary Fig. 8a,b). These data provide evidence of incomplete innervation of muscle fibres formed *de novo* from MuSCs in transplanted bioconstructs.

Re-innervation of muscle fibres in injured tissue and in transplanted muscle explants can take >30 days to occur^4^. For this reason, we chose to test for force production improvements at a later time point, 60 days after the treatment. Similar to 30-day time points, the results observed 60 days after treatment revealed that the bioconstruct show improved *ex vivo* but not *in vivo* force performances (Supplementary Fig. 9a). Consistently with *ex vivo* forces, immunohistochemistry analysis confirmed extensive regions of donor-derived myofibres in the transplanted regions (Supplementary Fig. 9b). These results demonstrate that *de novo* engineered muscle tissue persists in the host and restores forces *ex vivo* for up to at least 2 months. The fact that *in vivo* forces were still not restored even 2 months after the treatment indicates the need for additional therapeutic interventions to improve innervation.

Exercise has been proposed to improve muscle function following VML injuries^42^,^43^. Based on these suggestions, we hypothesized that a regime of exercise could further enhance the process of *de novo* myogenesis and innervation following the treatment of VML injuries with bioconstructs. First, we analysed the capacity of mice that received VML injuries to recover performances in voluntary running. Using computer-assisted wheels, we measured the time required for injured mice to return to the daily running habits measured for uninjured control mice. We found that following VML injury, it took 7 days to return to running these control distances (Supplementary Fig. 10a). Next, we tested the effect of running on mice that received VML injuries. We exercised these mice for seven days on a treadmill either immediately after the injury or after seven days of rest. We found that early exercise delayed muscle regeneration (smaller myofibre CSAs) and increased fibrosis (higher content of Collagen deposition), whereas later exercise instead accelerated myogenesis and reduced fibrotic formation (Supplementary Fig. 10b,c) Based on these results, we chose to begin exercise after 1 week of rest for mice with VML injuries. We imposed an exercise regimen on VML-injured mice that had received either no treatment or treatment with bioconstructs generated with eYFP^+^ MuSCs and eYFP^−^ MRCs. We exercised mice for 3 weeks on a treadmill for 60 min daily, and we compared then with non-exercised control groups.

**Figure 6:**
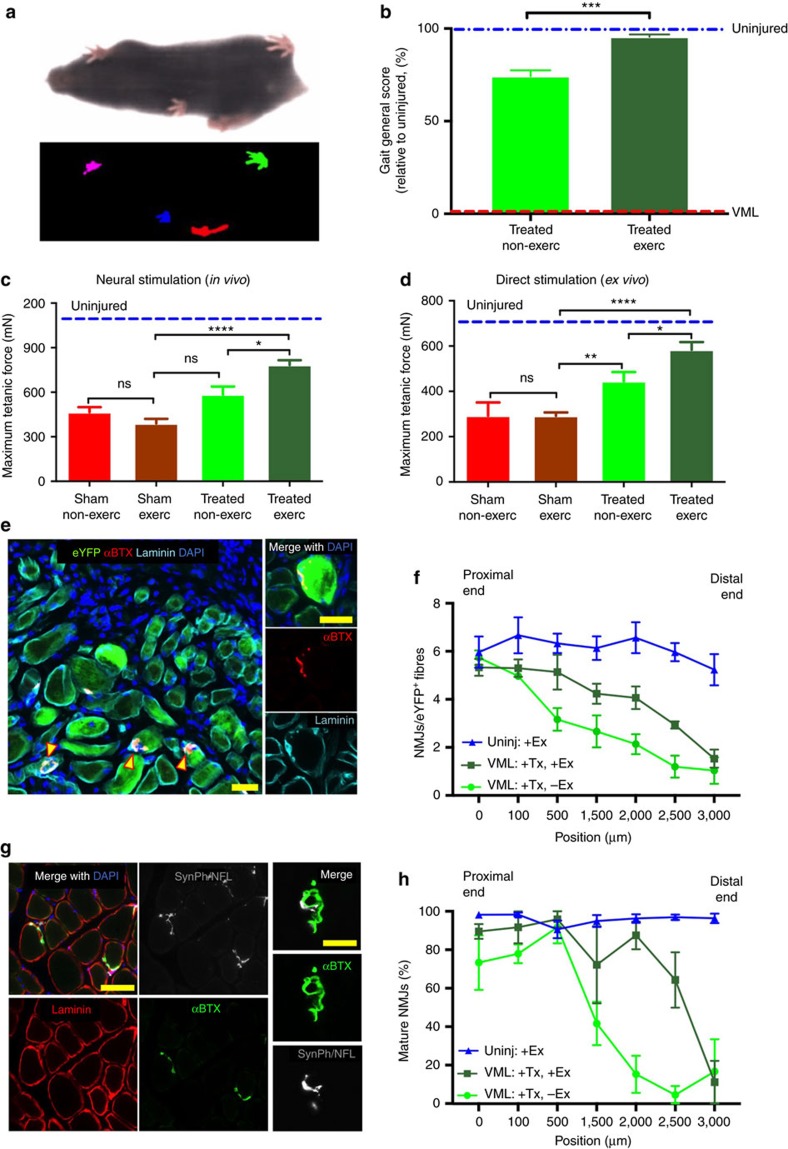
Exercise improves innervation of *de novo* myofibres and improves forces *in vivo.* (**a**) Representative image of a mouse during a gait analysis (top) and the gait footprints collected during the analysis (bottom). Mice were positioned in a transparent treadmill and a camera was positioned underneath to record the gait. (**b**) Quantification of the gait ‘disability score’ resulting from the analysis of 47 parameters (see Methods) (*n*=6). (**c**) *In vivo* force production measurements of TA muscles treated with bioconstructs following VML injury in non-exercised or exercised mice. After 30 days, the distal tendons were attached to a force transducer and contractions were induced through sciatic nerve stimulation (*n*=6). (**d**) *Ex vivo* force production measurements from non-exercised or exercised mice. The same muscles measured in **c** were then dissected and cultured in a chamber. The distal tendons were attached to a transducer and contractions were induced electrically in the culture bath (*n*=6). (**e**) (Left) Representative IF image of transplanted bioconstruct. Yellow arrows indicate donor-derived (eYFP^+^) myofibres with NMJs (αBTX^+^) within regions of the transplanted bioconstruct. (Right) Higher magnification of an NMJ associated with a donor-derived myofibre (scale bars=50 μm). (**f**) Quantification of NMJs in whole cross-sections of transplanted bioconstructs along 3 mm lengths of TA muscles. Muscles were either uninjured and exercised (‘-VML, +Ex’) or subjected to VML injury and bioconstruct treatment (‘+VML, +Tx’) without (‘-Ex’) or with (‘+Ex’) exercise (*n*=5). (**g**) (Larger panels) Representative IF images of myofibres with mature NMJs (αBTX^+^ and also stained positive for Synaptophysin (SynPh) and Neurofilament (NFL)) within regions of transplanted bioconstructs (scale bar=100 μm). (Smaller panels) Higher magnification of a mature NMJ (scale bar=10 μm). (**h**) Quantification of mature NMJs in whole cross-sections of uninjured muscles or of injured muscles with transplanted bioconstructs along 3 mm lengths of TA muscles characterized as in **f** (*n*=4). Data are±s.e.m. For statistical analysis, *t*-tests were used. **P<0.001; ****P<0.00001.

To test the functional deficits resulting from VML injuries, we performed gait analysis 30 days following the treatment of non-exercised or exercised mice. Using a scale in which untreated mice exhibited maximum deficit and uninjured mice showed no deficit, we found that treating VML with bioconstructs in non-exercised mice resulted in a functional recovery to ∼75% of that of uninjured mice (Fig. 6a,b). However, treatment with exercise resulted in a further improved score to ∼95% of the valued from uninjured mice (Fig. 6a,b). Next, we measured forces *in vivo* trough neural stimulation. Following exercise, muscles from exercised mice showed improved force production compared with muscles from non-exercised mice, showing a partial rescue of forces measured *in vivo* after VML injuries (Fig. 6c). Similarly, *ex vivo* force measurements also showed greater improvement in exercised mice (Fig. 6d). Moreover, despite similar muscle masses, treated muscles with VML injuries from mice that received exercise outperformed muscles from non-exercised mice in terms of force generation by either *in vivo* or *ex vivo* stimulation (Supplementary Fig. 11a). Taken together, these results show that exercise is capable of restoring forces *in vivo* of treated muscles with VML injuries.

We hypothesized that exercise was improving forces *in vivo* by ameliorating the deficit of innervation observed in non-exercised mice with VML injuries treated with bioconstructs. To test this, we performed immunohistochemistry on the same muscles analysed for force production. We first measured the number of NMJs associated with donor-derived myofibres within treated VML-injured TA muscles, from the proximal to the distal regions (Fig. 6e). Our results show that exercise increased the number of NMJs associated with donor-derived myofibres (eYFP^+^) across the muscle compared with muscles of non-exercised mice (Fig. 6f). Next, we measured the number of mature NMJs. These were characterized by the typical ‘pretzel-like’ morphology and positive immunostaining for Synaptophysin and Neurofilament, proteins expressed in the synaptic vesicles and the axons of the nerve, respectively (Fig. 6g). Again, exercise resulted in improved maturation of NMJs within transplanted muscles of exercised mice compared with muscles from non-exercised mice (Fig. 6h). The distribution of NMJs correlated with the *in vivo* forces in individual muscles. Muscles from non-exercised mice clustered in the lower end of the range, whereas muscles from exercised mice clustered in the upper end, indicating a shift toward larger masses and stronger *in vivo* forces with exercise (Supplementary Fig. 11b).

In addition, we asked whether exercise had any positive effect on vasculogenesis. Within transplanted bioconstructs regions, we quantified the average number of blood vessels per donor-derived myofibre and we found that exercise increased their number by twofold in regions with *de novo* myofibres (Supplementary Fig. 12a). Finally, we quantified the level of fibrosis and we found that exercise significantly reduced fibrosis compared with non-exercised mice (Supplementary Fig. 12b,c). Taken together, these results show that exercise improves the treatment of VML by reducing fibrosis and enhancing vascularisation.

### Bioconstructs partially repair chronic VML injuries

Chronic lesions represent the more common clinical presentations of VML injuries and are characterized by extensive muscle tissue remodelling and scar formation^1^,^2^,^3^,^44^. To test if our MuSC^+^/MRC^+^ bioconstructs treatment strategy was also capable of partially restoring muscle structure and function in chronic lesions, we induced VML lesions and waited 30 days before transplanting MuSC^+^/MRC^+^ bioconstructs. We then waited another 30 days and assessed the biomechanical and histological outcomes. Assessment of muscle force generation *in vivo* showed, as with the acute treatments, no significant increase compared with controls (scar tissue debridement but no bioconstruct treatment) (Supplementary Fig. 13). However, *ex vivo* stimulation of treated muscles showed a partial recovery of force production when compared with controls (Supplementary Fig. 13), suggesting that even chronic VML lesions may be amenable to treatment using our MuSC bioconstructs approach.

Immunohistochemical analyses showed local areas of donor-derived myofibres with few NMJs (Supplementary Fig. 15). Morphometric analysis revealed no significant difference in *de novo* myofibre CSA compared with fibres generated in VML injuries treated acutely (Supplementary Fig. 15a). However, despite myofibre CSAs that were similar to the acute model (Supplementary Fig. 15a), there were significantly fewer eYFP^+^ myofibres in the chronic model (Supplementary Fig. 15b), consistent with smaller force improvements after treatments (Supplementary Fig. 13). These results show that bioconstructs generated with MuSCs and MRCs can be employed to treat chronic VML injuries, partially restoring structure and function.

### Bioconstructs made of human MuSCs and MRCs

**Figure 7:**
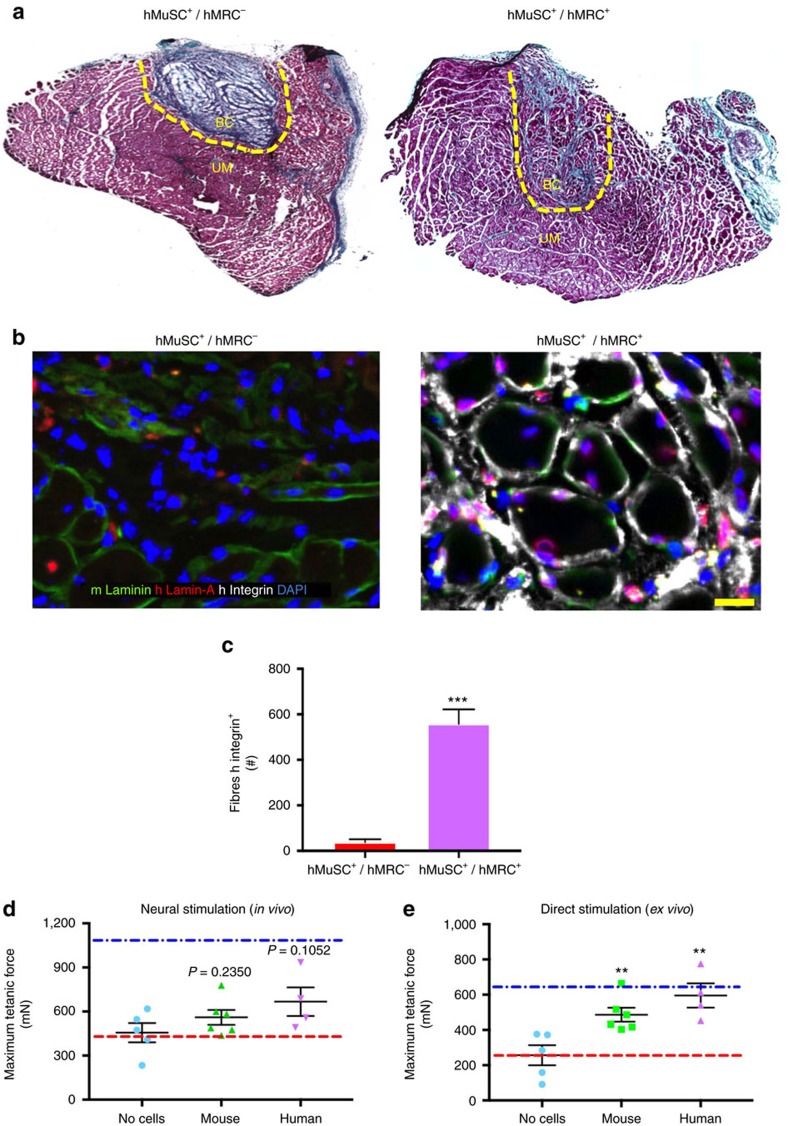
Human MuSCs and MRCs generate *de novo* human muscle tissue in VML injuries. (**a**) Representative histological images of murine TA muscles following VML injury and treatment with hMuSC/hMRC bioconstructs. The yellow dashed lines indicate the regions where bioconstructs were transplanted (scale bar=2 mm). (**b**) Representative IF staining of mouse and human proteins in the same muscles as in **a** (scale bar=50 μm). (**c**) Quantification of the number of human Integrin α7β1^+^ myofibres in muscles treated with bioconstructs generated with either human MuSCs alone or together with human MRCs (*n*=4). (**d**) Bioconstructs were generated with: no cells, freshly isolated mouse MuSCs and MRCs, or freshly isolated human MuSCs and MRCs. These bioconstructs were transplanted in VML injuries of TA muscles of immunocompromised mice. Muscles were analysed for force production 30 days later. The graph shows quantification of *in vivo* force production comparing the three conditions. (**e**) The same muscles analysed in **d** were analysed and quantified for *ex vivo* force production. Data are±s.e.m. For statistical analysis, *t*-tests were used. **P<0.001.

Designing treatments for VML injuries in mice is an important step to treating similar injuries in humans. With this translational goal in mind, we wanted to test whether transplantation of human MuSC^+^/MRC^+^ bioconstructs would be as effective as murine MuSC^+^/MRC^+^ bioconstructs. We thus used FACS to isolate human MuSCs and MRCs from operative samples obtained from consenting patients. We then generated human MuSC^+^/MRC^+^ and MuSC^+^/MRC^−^(which we designate as hMuSC^+^/hMRC^+^ and hMuSC^+^/hMRC^−^, respectively) bioconstructs according to the procedures identical to those done with mouse cells. We transplanted these bioconstructs into immunodeficient NOD/scid IL2Rgnull (NOD.Cg-*Prkdc*^*scid*^ *Il2rg*^*tm1Wjl*^/SzJ or NSG) mice with acute VML injuries. After 30 days, we assessed human myofibre formation within the muscles. Histochemical analysis showed that treatment with the hMuSC^+^/hMRC^+^ bioconstructs resulted in muscle tissue formation and minimal fibrotic infiltration (Fig. 7a). By contrast, transplanted MuSC^+^/MRC^−^ bioconstructs resulted in negligible *de novo* muscle formation (Fig. 7a). Instead, the transplanted area appeared to consist primarily of residual scaffold, appearing as a fibrotic scar.

Immunohistochemical analysis revealed that hMuSC^+^/hMRC^+^-treated muscles contained myofibres within the regions of the transplanted bioconstructs that stained positively for human Integrin α7β1 (Fig. 7b). Quantification of muscles transplanted with hMuSC^+^/hMRC^−^ bioconstructs revealed rare Integrin α7β1^+^ myofibres within the region of the transplant (Fig. 7c). Conversely, muscles treated with hMuSC^+^/hMRC^+^ bioconstructs resulted in numbers of donor-derived myofibres comparable to those observed following treatment with murine cells (Fig. 7c; compared with Fig. 5f). Moreover, immunostaining of human Lamin-A, a perinuclear protein, revealed positive nuclei across the regions of the transplanted hMuSC^+^/hMRC^+^ bioconstructs (Fig. 7b). Only a limited number of Lamin-A^+^ human nuclei were visible in muscles treated with the hMuSC^+^/hMRC^−^ bioconstructs. We then tested force production of muscles treated with hMuSC^+^/hMRC^+^ bioconstructs after 30 days and found that, as with bioconstructs containing murine cells, *in vivo* forces did not improve significantly but *ex vivo* forces did (Fig. 7d,e). Together, these results demonstrate that hMuSCs can contribute to myofibre generation in and functional restoration of VML injuries, suggesting the possibility of using these bioconstructs in a scalable approach to treat VML injuries in humans.

## Discussion

VML is an injury of the skeletal muscle caused by major trauma, such as battle wounds or tumour excision^1^,^2^. Endogenous MuSCs, despite their remarkable regenerative capacity, are unable to restore the muscle structure and function compromised by VML injuries^12^,^40^. Current treatments are limited to scar tissue debridement and autologous tissue transfer at the site of tissue defect^4^,^6^. By combining decellularized muscle tissue scaffolds with freshly isolated MuSCs and MRCs, we were able to generate bioconstructs that, when transplanted in irrecoverable acute VML injuries in a murine model, resulted in *de novo* myofibre formation *in vivo*. Moreover, transplanted bioconstructs were able to partially restore structure and *ex vivo* force production, but insufficient innervation of the *de novo* myofibres. However, when treatment was followed by physical therapy, innervation was improved and *in vivo* forces were restored. Similarly, chronic VML injuries, a challenging clinical condition complicated by severe fibrosis and tissue remodelling, responded positively to our treatment. We were also able to repair and restore muscle structure and function by the transplantation of bioconstructs established with human MuSCs and MRCs, highlighting the translational potential of this approach. These results, a product of our combined bioengineering and stem cell technologies, have important implications in the development of future stem cell-based therapies for the treatment of VML injuries.

The use of ECM-based biomaterials to treat VML injury has yielded variable results^45^,^46^. To date, these treatments have shown some degree of myofibre regeneration with limited improved force production in animal models^19^. It has been suggested that the application of ECM proteins and factors derived from decellularized tissues might act as biochemical stimuli, capable of inducing tissue regeneration^15^, but this remains to be demonstrated. When structure and function are too severely compromised, as is the case in most VML injuries, ECM-based materials alone cannot effectively rescue the tissue but can result instead in significant fibrotic formation that limits efficacy of these treatments^18^,^47^.

An alternative strategy that combines myogenic cells with scaffolds has been shown to improve the efficacy of scaffolds alone. Engrafting cultured myogenic cells in decellularized scaffolds prior to transplantation in animal models results in *de novo* muscle fibre formation that persists in the host following transplantation into VML injuries contributing, at least partially, to muscle structure and force production^16^,^23^,^48^. However, cultured cells show limited efficacy, compared with freshly isolated stem cells, in their ability to regenerate tissues^9^,^49^.

Recently, VML treatments utilising minced muscle preparations, including all of the MRCs, have yielded promising results, including partial structural and functional recovery^28^,^50^. Our results showed that treating VML with bioconstructs containing only MuSCs had a limited capacity to restore tissue structure and function. Conversely, when we transplanted bioconstructs generated with MuSCs and MRCs, we observed remarkably improved force production and partial regeneration of structure. Among the multiple MRC populations, we investigated the particular role of ECs and found that they are necessary and sufficient to sustain MuSC expansion and engraftment within transplanted bioconstructs. These ECs were able to engraft and participate in *de novo* vasculogenesis within transplanted bioconstructs. Other MRCs may also have critical roles. Within the HCs, different CD45^+^ populations can be stimulated by implanted biomaterials within traumatic injuries^51^. It was recently shown that decellularized ECM scaffolds implanted in muscles with VML injuries can modulate the immune microenvironment through a mTOR/Rictor-dependent T helper 2 pathway that guides interleukin-4-dependent macrophage polarisation, an important process for functional muscle recovery^51^.

Our results show an average of >400 myofibres generated *de novo* per transplanted bioconstruct (for example, Fig. 6e). A single murine myofibre of a lower limb muscles generates an average of 0.5 mN absolute force^52^. Our *ex vivo* results, which showed an average of 250 mN force rescue per leg (Fig. 5c), are consistent with these data. These improvements in force production were contrasted by persisting anatomical deficits. Although a clear improvement over untreated VML injuries, our treatment showed lingering signs of fibrosis and irregular myofibre morphology. Continued optimisations of our bioconstruct formulation and transplantation processes, for example, using more refined MRC-based treatments, might further improve tissue reconstruction following VML.

Our measurements of force production *in vivo* did not show nearly the improvements in force generation as those observed when muscles were stimulated *ex vivo* (for example, Fig. 5b versus Fig. 5c). We hypothesized that this discrepancy might be explained by inefficient innervation of the *de novo* muscle engineered within the transplanted bioconstructs. Immunohistochemical assessment within the regions of the transplanted bioconstructs showed that only a limited number of myofibres stained positive for mature NMJs 30 days following treatment (Supplementary Fig. 8a,b). As it can take >2 months for damaged muscle to fully re-innervate^53^, it is reasonable to expect improved innervation at later times points. However, when we examined muscles 60 days following treatment, *in vivo* force production did not outperform untreated muscles. It might be possible to further improve innervation by supporting the bioconstruct treatments with exercise^42^,^43^. There is evidence that aggressive exercise immediately after surgical implantation of ECM scaffolds to VML injuries has significant benefit on matrix remodelling^14^,^54^. Moreover, positive effects of exercise have been observed within 30 days following muscle grafting in rats, resulting in improved re-innervation^53^,^54^,^55^. In agreement with these reports, when we followed treatment of VML injuries with bioconstructs and exercise, we found enhanced maturation of innervation and enhanced force production *in vivo* at 30 days following treatment. Moreover, we showed that exercise increases vascularisation and reduces fibrosis of *de novo* tissues engineered in VML injuries. This evidence indicates that exercise enhances the regenerative potential of VML treatments by helping to overcome limitations in current methods, such as limited innervation and excessive fibrosis. Alternative strategies might include delivering neurogenic factors within the bioconstructs^56^.

Moving forward, the availability of MuSCs will need to be addressed to regenerate large muscle volumes. As noted above, expanding MuSCs *in vitro* results in a loss of stem cell potency, ultimately limiting their value for therapeutic use^9^,^49^,^57^. Recently, however, new strategies to preserve cultured MuSC function and potency have been proposed^49^,^58^. These strategies are based on recreating the physiological MuSC microenvironment, or niche, to mimic the biochemical, molecular and/or biophysical conditions through which potency is maintained^26^,^49^,^59^. The most common approaches, which include addition of factors or small molecules to the growth media and recreation of the physiological substrate stiffness on which cells are cultured, have demonstrated that it is possible to preserve, at least partially, the original ‘stemness’ of mouse and human MuSCs, renewing their candidacy for use in transplantation after prolonged periods in culture^26^,^48^,^59^,^60^. Similar strategies could be adopted to expand freshly isolated MRCs.

Our findings represent an important step forward in validating the therapeutic potential of stem cell-based treatments in engineering tissues capable of treating VML injuries and, more generally, in muscle reconstruction. Although there is certainly a need for a substantial scaling aimed for treatment of VML injuries of large muscles, other important translational applications include the reconstruction of congenitally defective or injured small human muscles, such as cleft lip muscles or eye muscles. These aspects represent clinical conditions that could readily benefit from treatment strategies such as those described here. We demonstrate the translational potential of this reconstructive tissue engineering strategy by replicating our results using human MuSCs and MRCs, offering the exciting possibility that these therapeutic strategies might be readily translated to a clinical setting.

## Methods

### Animals

C57BL/6, ROSA26^eYFP^ and FVB-Tg(CAG-luc,-GFP)L2G85Chco/J male mice were obtained from Jackson Laboratory. NOD/MrkBomTac-*Prkdc*^*scid*^ female mice were obtained from Taconic Biosciences. Pax7Cre^ER^ mouse were provided by Dr Charles Keller, Oregon Health and Science University, Portland, OR, USA^61^. Tamoxifen injections for Cre recombinase activation were performed administering five doses (5 mg per mouse) every 2 days and waiting a minimum of 7 days before using the animals experimentally^61^. To control for tamoxifen injection toxicity, we injected all mice with tamoxifen. Mice were housed and maintained in the Veterinary Medical Unit at the Veterans Affairs Palo Alto Health Care Systems. Animal protocols were approved by the Administrative Panel on Laboratory Animal Care of Stanford University.

### Human skeletal muscle specimens

Subjects ranged in age from 51 to 75 years. The human muscle biopsy specimens were taken after patients gave informed consent, as part of a human studies research protocol which was approved by the Stanford University Institutional Review Board. All experiments were performed using fresh muscle specimens, according to availability of the clinical procedures. Sample processing for cell analysis began within one hour of specimen isolation. In all studies, standard deviation reflects variability in data derived from studies using true biological replicates (that is, unique donors). Data were not correlated with donor identity.

### Ablation model

NOD/SCID or NSG mice of ages 3 months (chronic study) or 4 months (acute study) were placed under 2.5% isoflurane anaesthesia for surgical procedures. An incision was made directly over the TA muscle and was extended proximally up to the patellar tendon and distally to the myotendinous junction of the TA muscle. The thin layer of fascia covering the TA muscle was dissected away and a 2 × 7 mm stencil was placed on the muscle. A scalpel was used to make a 2 mm deep cut around the stencil removing a 2 × 7 × 2 mm piece of the muscle and creating ∼40% ablation of the TA muscle. The excised muscle was weighed to confirm the weight to be 15±1.5 mg. If no immediate transplant was to be performed, the incision was sutured closed and Buprenorphine (0.1 mg kg^−1^) and Baytril (5 mg kg^−1^) were administered.

### MRC Isolation and purification

Muscles were harvested from hind limbs and mechanically dissociated to yield a fragmented muscle suspension using a gentleMACS dissociator (Miltenyl Biotec). This was followed by a 90 min digestion in a Collagenase II-Ham’s F10 solution (500 units per ml; Invitrogen). After washing, a second digestion was performed for 30 min with Collagenase II (100 units per ml) and Dispase (2 units per ml; Invitrogen). The resulting cell suspension was washed, filtered and stained with VCAM-biotin (clone 429; BD Bioscience), CD31-APC (clone MEC 13.3; BD Bioscience), CD45-APC (clone 30-F11; BD Bioscience) and Sca-1-Pacific-Blue (clone D7; Biolegend) antibodies at a dilution of 1:75. Streptavidin-PE-cy7 was used to amplify the VCAM signal (BD Biosciences, 1:75) and FACS grade DAPI dilactate for non-viable cell exclusion (D3571 Invitrogen). For Pax7Cre^ER^/ROSA26^eYFP^ mice, MuSCs were directly sorted by endogenous eYFP protein expression. Human MuSCs were purified from fresh operative samples^60^,^61^,^62^. Operative samples were carefully dissected from adipose and fibrotic tissue and a disassociated muscle suspension prepared as described for mouse tissue. The resulting cell suspension was then washed, filtered and stained with anti-CD31-Alexa Fluor 488 (clone WM59; BioLegend; #303110, 1:75), anti-CD45-Alexa Fluor 488 (clone HI30; Invitrogen; #MHCD4520, 1:75), anti-CD34-FITC (clone 581; BioLegend; #343503, 1:75), anti-CD29-APC (clone TS2/16; BioLegend; #303008, 1:75) and anti-NCAM-Biotin (clone HCD56; BioLegend; #318319, 1:75). Unbound primary antibodies were then washed and the cells incubated for 15 min at 4 °C in streptavidin-PE/Cy7 (BioLegend) to detect NCAM-biotin. Cell sorting was performed on calibrated BD-FACS Aria II or BD FACSAria III flow cytometers equipped with 488-nm, 633-nm and 405-nm lasers to obtain the MuSC population. A small fraction of sorted cells was plated and stained for Pax7 and MyoD to assess the purity of the sorted population. For the FACS gating strategy, see Supplementary Information.

### Decellularized scaffold preparation

ECM-based scaffolds for use as bioconstructs were generated form decellularized muscle tissue as follows. Lower limbs of C57BL/6 mice were dissected such that both the proximal and distal ends of the TA muscles remained in mechanical tension by their natural attachment points. The lower limbs were then placed in a decellularization buffer (1% sodium dodecyl sulfate, Sigma B01587, in phosphate-buffered saline (PBS) solution) and allowed to react for 3 days, then rinsed in fresh PBS every 24 h for 5 days. The resulting decellularized TAs were then dissected from the bone and transferred into the tubular culture chambers of the bioreactor (made of Tygon E-3603 biopolymer), where they were held in place with stainless steel minutien pins (FST, 0.2 diameter, 10 mm length, AM00030).

### Bioconstruct preparation

Scaffold preparation began with loading our isolated cells and ECM-based hydrogels into insulin syringes. These syringes were then positioned on a SP220I syringe pump (World Precision Instruments; Sarasota, FL, USA) to allow precise control over the flow rate of the injections. Freshly purified cells were mixed in ECM-based hydrogel solution at the ratios of: 150,000 MuSCs; 100,000 HCs; 140,000 ECs; 40,000 FAPs; 70,000 fibroblast-like cells. Evans Blue dye was added to the cell solution allowing visualization of the injected regions. A total volume of 15 μl was loaded into insulin syringes with fresh reconstitution solution, and mounted on a syringe pump. The decellularized scaffold, pinned inside the tubular culture chambers, was mounted on a micromanipulator and positioned in front of the syringe needle. The needle was then carefully driven through the decellularized scaffold, and 20 μl of the cell per hydrogel solution was deposited at a flow rate 1.5 μl min^−1^. To ensure that cells were distributed evenly throughout the entire scaffold, the needle was retracted from the scaffold at a rate of 500 μm every 30 s. The resulting bioconstructs, still contained within the tubular culture chamber, were then transferred into an incubator set at 37 °C/5% CO_2_. Each culture chamber was connected to a bioreactor to allow perfusion with fresh media while the hydrogel cured.

### Bioreactor design

The bioreactor was designed to sustain the 3D cell culture within each bioconstruct by perfusing it with fresh culture media. The bioreactor was made of four tubing holders, connected by luer-lock adapters to ¼ inch Tygon E-3603 biopolymer tubular culture chamber that were epoxied to pulley wheels and connected to swivel tubing adaptors. The swivel tubing adaptors let fluid flow freely through the system, while allowing internal rotation. The tubing employed for the circulation of media was made of gas permeable silicon tubing (Masterflex BioPharm Plus platinum-cured silicone pump tubing, L/S 16, 25 ft) organized in a serpentine pattern to allow optimal media oxygenation and gas exchange. Circulation of media was controlled by an automatic pump (Geniethouch, Lucca Technologies), at a flow rate of 1 ml min^−1^. Bioconstructs were positioned in the bioreactor culture tubular chambers before starting the reconstitution with hydrogels and cells. The chambers were then immediately connected to the tubing of the bioreactors after reconstitution, and the recirculation was started 30 min after the curing of the hydrogel and initiating the gelation process. Each bioconstruct remained in the bioreactor for not longer than 2 h before being transplanted.

### Bioconstruct transplantation

For the acute VML model, bioconstructs were transplanted immediately after the ablation surgery was performed (*n*=6). The bioconstructs were inserted directly into the injury site and were sutured into place at both the proximal and distal ends using 10–0 sutures (Vivryl V966G, Ethicon). The intact host muscle tissue was then folded over the top of the transplant and sutured in place to optimize coverage of the scaffold. The skin incision was then sutured closed and Buprenorphine (0.1 mg kg^−1^) and Baytril (5 mg kg^−1^) were administered. For the chronic VML model (*n*=6), an incision was made over the scar and a debridement surgery was performed to remove the scar tissue. Transplantation of the bioconstructs was then performed similar to acute injuries. Sham controls received incision and debridement but not bioconstruct transplantation.

### Voluntary running

Each subject was singly housed in cage with computer-controlled running wheels. All animals had access ad libitum to food, water and wheel running. Prior to the experimental period, subjects were given 3 days to acclimate to the cages and their surroundings. At day 3, the experimental group (*n*=4) had internal ablations performed on both legs as described in ‘Ablation Model’ section in ‘Methods’. Mice were then returned to their cages with computer-controlled running wheels and allowed to run freely. Non-exercised controls were singly housed in cages with locked wheels. Cycles of 12-h day per night light were used in the rooms housing the mice and their cage bedding was changed once a week. Each cage wheel was attached to a counter motor that was then attached to data acquisition software that recorded the number of rotations that occurred during each 15 min interval for 2 weeks.

### Treadmill running

Before each mouse began its exercise programme, it was acclimated to the Exer 3/6 treadmill (Columbus Instruments) for 3 days with a 10 min running period at 10 m min^−1^ each day (*n*=6). At the start of each training regimen, the mice slowly acclimated to the speed of the treadmill by beginning at 7 m min^−1^ and increasing the speed at 0.5 m min^−1^ until a speed of 12 m min^−1^ was reached. The mice then ran progressively longer each day for 7 days at a speed of 15 m min^−1^ for 3 weeks. A low voltage was applied to a grate at the end of the treadmill to encourage the mice to continue running for the duration of the protocol.

### Gait analysis

Gait capture and analysis was performed using a Digigait System (Mouse Specifics). Before videos were recorded, mice were allowed to acclimate in the run chamber for 5 min and then ran at 10 cm s^−1^ for another 5 min to acclimate to belt movement. Once the mouse was determined to be comfortable, the brightness, contrast and viewing frame were adjusted so that the paws could be easily seen within the captured images. The belt speed was then increased to 20 cm s^−1^ and recording of the gait was immediately begun. Videos were recorded for as long as it took to obtain at least three segments of 3 s of uninterrupted running (running in which the mouse did not stop, jump or put its paws on the walls of the chamber). Run videos were captured on a flat surface and at a 10 degree incline. Once the segments were obtained, they were post-processed and analysed by the software provided with the Digigait imaging system. Each segment was individually adjusted with a binary threshold adjustment tool to remove any noise and to establish well defined paw areas. Data were manually adjusted to remove any artifacts in the paw area-plots the system included in the results. Once the data had been checked for accuracy, they were inserted into a MATLAB programme to sort through the parameters, perform statistical comparisons and return an overall gait score.

### *In vivo* force production

Mice were placed under 2.5% isoflurane anaesthesia to perform the TA isolation surgery. A 2-inch incision was made from the iliac crest distally along the thigh to begin the process of exposing the sciatic nerve. A landmark fat pad was then cut, allowing separation of the cranial thigh muscles and access to the sciatic nerve. The fascia surrounding the nerve was dissected away and a small piece of Parafilm was placed under the nerve for later placement of the stimulatory nerve cuff. Another incision was made from the patellar tendon down to the myotendinous sheath located in the ankle to ensure complete TA muscle exposure. Saline was added throughout the procedure to ensure the tissue remained hydrated. Using a surgical microscope, the myotendinous sheath was cut to free the distal tendon of the TA for manipulation. The TA muscle was then carefully separated from the surrounding muscles using a blunt dissection technique. The medial fascia between the TA muscle and the tibia was carefully cut away up until the point where fibres began inserting into the bone (where the tibia begins to bow inward). On the lateral side, the fascia between the TA muscle and the other lower-limb muscles was dissected away and any additional muscle groups separated to ensure they did not contribute to force production. The distal end of the TA tendon was superglued between two flat bars, providing a leverage point past which a knot could not slip during contraction events. The TA tendon was then cut distal to the bars to free up the muscle for subsequent force production. An Aurora Scientific 1300-A Whole Mouse Test System was used to gather force production data. The mouse was placed on the stage and a pin was placed through an L bracket behind the patellar tendon to secure the knee joint and ensure isometric muscle contractions. The ankle of the mouse was also secured using tape to ensure the leg remained in the same position throughout the testing procedure. At one end a knot was tied around the distal tendon, secured by the affixed toothpicks, and at the other it was affixed to the muscle lever. The lever was positioned such that it was perpendicular to the TA muscle. A small, two wire, bipolar nerve cuff was then positioned around the sciatic nerve in such a way that both electrodes were touching the nerve. The previously placed Parafilm strip helped to ensure that unwanted stimulation of the surrounding tissue did not occur.

### Data acquisition

Calibrated Dynamic Muscle Control and Analysis Software (Aurora Scientific) and a 701C Stimulator (Aurora Scientific) were used to acquire muscle force measurements. First, the rough optimal voltage was found by using course voltage adjustments and a manual stimulation on the 701C stimulator to determine what voltage yielded the most powerful twitches. Then, Force–Length measurements were obtained by stretching the TA muscle and measuring the twitch forces produced at 0.5 mm increments using six electrical pulses of 1 ms each at 1 Hz. When performing twitch tests a 1-minute rest period was allowed between each electrical pulse. Once the optimal length (stretched length at which the highest twitch force was observed) was determined, a more rigorous voltage recruitment analysis was performed by measuring the twitch force produced at 1 volt intervals from 1 to 10 volts using the same twitch protocol as above. If the twitch force was found to still be rising at 10 volts, testing was performed until the values plateaued. Having determined the optimal length and voltage, a 500 ms tetanic stimulation at 100 Hz was performed to ensure the knot and force output remained stable during tetanic stimulation. Once validated, a tetanic stimulation frequency sweep from 60 to 140 Hz at 20 Hz intervals was performed to determine the maximum tetanic force produced by the muscle. A rest period of 2 min was given between each tetanic stimulation. Imaging was performed in blinded fashion: the investigators performing the imaging did not know the identity of the experimental conditions for the transplanted cells.

### *Ex vivo* force production

An 800A *in vitro* apparatus (Aurora Scientific) was used to perform the *ex vivo* testing of the TA muscles. The testing chamber, along with the Krebs solution (Sigma), was kept at 25 °C using an external water heater. To help ensure tissue viability, a 95% O_2_, 5% CO_2_ mixture was used to oxygenate the testing chamber along with a petri dish containing Krebs solution. Mice were killed and the lower limbs excised from the ankle to ∼0.5 inches above the patellar tendon to ensure the entire TA muscle was removed. The legs were then pinned down in the petri dish coated with an elastomer (Sylgard-184, Dow Corning). The TA muscles were carefully dissected from the legs under a microscope and held under tension until attached to the force transducer lever. The muscle was attached to the force transducer lever by directly tying a knot around the top of the patellar tendon. The TA muscle was then attached to the force transducer lever and the bottom of the testing apparatus to secure the TA muscle in place. The testing chamber was raised around it to immerse the muscle in Krebs solution. A similar testing procedure to the *in vivo* protocol was used *ex vivo* except no voltage recruitment testing was performed. Imaging was performed in blind: the investigators performing the imaging did not know the identity of the experimental conditions for the transplanted cells.

### Bioluminescence imaging

Bioluminescent imaging was performed using the Xenogen IVIS-Spectrum System (Caliper Life Sciences). Mice were anaesthetized using 2% isoflurane at a flow rate of 2.5 l min^−1^ (*n*=4). Intraperitoneal injection of D-Luciferin (50 mg ml^−1^, Biosynth International Inc.) dissolved in sterile PBS was administered. Immediately following the injection, mice were imaged for 30 s at maximum sensitivity (f-stop 1) at the highest resolution (small binning). Every minute a 30 s exposure was taken, until the peak intensity of the bioluminescent signal began to diminish. Each image was saved for subsequent analysis. Imaging was performed in blinded fashion: the investigators performing the imaging did not know the identity of the experimental conditions for the transplanted cells.

### Bioluminescence image analysis

Analysis of each image was performed using Living Image Software, version 4.0 (Caliper Life Sciences). A manually-generated circle was placed on top of the region of interest and resized to completely surround the limb or the specified region on the recipient mouse. Similarly, a background region of interest was placed on a region of a mouse outside the transplanted leg.

### Functional vascularisation assessment

Mice received a tail vein injection of 200 μm of biotinylated-Isolectin diluted in saline solution at a concentration of 2 mg ml^−1^ (*n*=3). Tail veins were dilated prior injection by heat using a lamp while mice were awake. Each injection was performed using a sterile 24-gauge needle and a sterile 1 ml syringe. Following the injections, mice were single caged for 10 min before being killed to collect the muscles for analysis.

### Tissue harvesting

TA muscles were carefully dissected away from the bone, weighed and placed into a 0.5% PFA solution for fixation overnight. The muscles were then moved to a 20% sucrose solution for 3 h or until muscles reached their saturation point and began to sink. The tissues were then embedded and frozen in optimal cutting temperature medium and stored at −80 °C until sectioning. Sectioning was performed on a Leica CM3050S cryostat that was set to generate 10 μm sections. Sections were mounted on Fisherbrand Colorfrost slides. These slides were stored at −20 °C until immunohistochemistry could be performed.

### Histology

TA muscles were fixed for 5 h using 0.5% electron-microscopy-grade paraformaldehyde and subsequently transferred to 20% sucrose overnight. Muscles were then frozen in optimal cutting temperature, cryosectioned at a thickness of 10 μm and stained. For colorimetric staining with Hematoxylin and Eosin (Sigma) or Gomorri Trichrome (Richard-Allan Scientific) samples were processed according to the manufacturer’s recommended protocols.

### Immunostaining

A 1 h blocking step with 20% donkey serum/0.3% Triton in PBS was used to prevent unwanted primary antibody binding for all samples. Primary antibodies were applied and allowed to incubate over night at 4 °C in 20% donkey serum/0.3% Triton in PBS. After four washes with 0.3% PBST, fluorescently conjugated secondary antibodies were added and incubated at room temperature for 1 h in 0.3% PBST. After three additional rinses each slide was mounted using Fluoview mounting media.

### Antibodies

The following antibodies were used in this study. The source of each antibody is indicated. Mouse: eMHC (DSHB, #F1.652, 1:50); CD31 (BD Pharmingen, #553371, 1:100); GFP (Invitrogen, #A11122, 1:250); Luciferase (Sigma-Aldrich, #L0159, 1:200); Collagen I (Cedarlane Labs, #CL50151AP, 1:200); HSP47 (Abcam, #ab77609, 1:200); α-Bungarotoxin (Life Technologies, # B-1196, 1:500); Laminin (Millipore, #MAB1903, 1:750); Synaptophysin (Abcam, #ab32594, 1:300); Neurofilament (Life Technologies, #MA5-14981, 1:200). Human: Lamin-A (Abcam, #ab108595, 1:200); Integrin (AbD Serotec, # MCA699PE, 1:200). Secondary antibodies (AlexaFluor, Invitrogen,1:1,000). Biotinylated GSL I-IsolectinB4 (Vector Laboratories, #B-1205).

### Hydrogels

All hydrogels were generated according to the manufacturer. Each muscle-derived ECM-based hydrogel was generated as follows: skeletal muscle tissue was decellularized with a 1% sodium dodecyl sulfate solution, made by adding appropriate volumes of 20 × PBS, 10 × sodium dodecyl sulfate, and ultrapure water. Rinses and solution changes were repeated every 24 h until the tissue was completely white (usually 3–4 days). Isopropyl alcohol was used to remove the presence of lipids after decellularization to prevent inhibition during subsequent gelation of the digested material. Frozen ECM was lyophilized and milled (Wiley Mini-Mill, #40 or #60 filter) to generate a particulate for subsequent protease digestion. In order to liquefy the ECM, milled ECM was partially digested in pepsin. After 48 h, the liquid ECM was titrated to pH 7.4, 1 × PBS and a final material concentration of 6 mg ECM per ml^−1^. Material was then aliquoted, frozen and lyophilized for long-term storage for up to 1 year at −80 °C. When ready for use, lyophilized ECM was resuspended in an equivalent volume of sterile water for use. Details are presented in Table 1.

### Imaging

Samples were imaged using standard fluorescent microscopy and either a × 10 or × 20 air objective. Volocity imaging software was used to adjust excitation and emission filters and came with pre-programmed AlexaFluor filter settings, which were used whenever possible. All exposure times were optimized during the first round of imaging and then kept constant through all subsequent imaging.

### Image analysis

Image J was used to calculate the percentage of area composed of Collagen by using the colour threshold plugin to create a mask of only the area positive for Collagen. That area was then divided over the total area of the sample which was found using the free draw tool. All other analyses were performed using Volocity software and manually counting fibres using the free draw tool and also counting the number of nuclei, eMHC^+^ fibres, NMJs and blood vessels by hand.

### Lentiviral transduction

Luciferase and GFP protein reporters were subcloned into a third generation HIV-1 lentiviral vector (CD51X DPS, SystemBio). To transduce freshly isolated MuSCs cells were plated at a density of 200,000 cells per well on a six-well plate and were incubated with 60 μl of concentrated virus and 8 μl polybrene. Plates were spun for 5 min at 3,200 g, and for 1 h at 2,500 g at 25 **°**C. Cells were then washed with fresh media two times, scraped from plates, spun down and resuspended in the final volume according to the experimental conditions.

### Statistical analysis

Unless otherwise noted, all statistical analyses were performed using GraphPad Prism 5 (GraphPad Software). For statistical analysis, *t*-tests were used. All error bars represent s.e.m.; *P<0.05; **P<0.001; ***P<0.0001.

### Data availability

The data that support the findings of this study are available from the corresponding author upon reasonable request.

## Additional information

**How to cite this article:** Quarta, M. *et al*. Bioengineered constructs combined with exercise enhance stem cell-mediated treatment of volumetric muscle loss. *Nat. Commun.* **8,** 15613 doi: 10.1038/ncomms15613 (2017).

**Publisher’s note:** Springer Nature remains neutral with regard to jurisdictional claims in published maps and institutional affiliations.

## Supplementary Material

Supplementary Information Supplementary Figures and Supplementary Table.
