# Supplementary material for: Bioengineered constructs combined with exercise enhance stem cell-mediated treatment of volumetric muscle loss
Source: Nat Commun. 2017 Jun 20;8:15613. doi: 10.1038/ncomms15613 (PMC5481841; doi:10.1038/ncomms15613)
Supplement: Supplementary Information — Supplementary Figures and Supplementary Table. [file ncomms15613-s1.pdf]

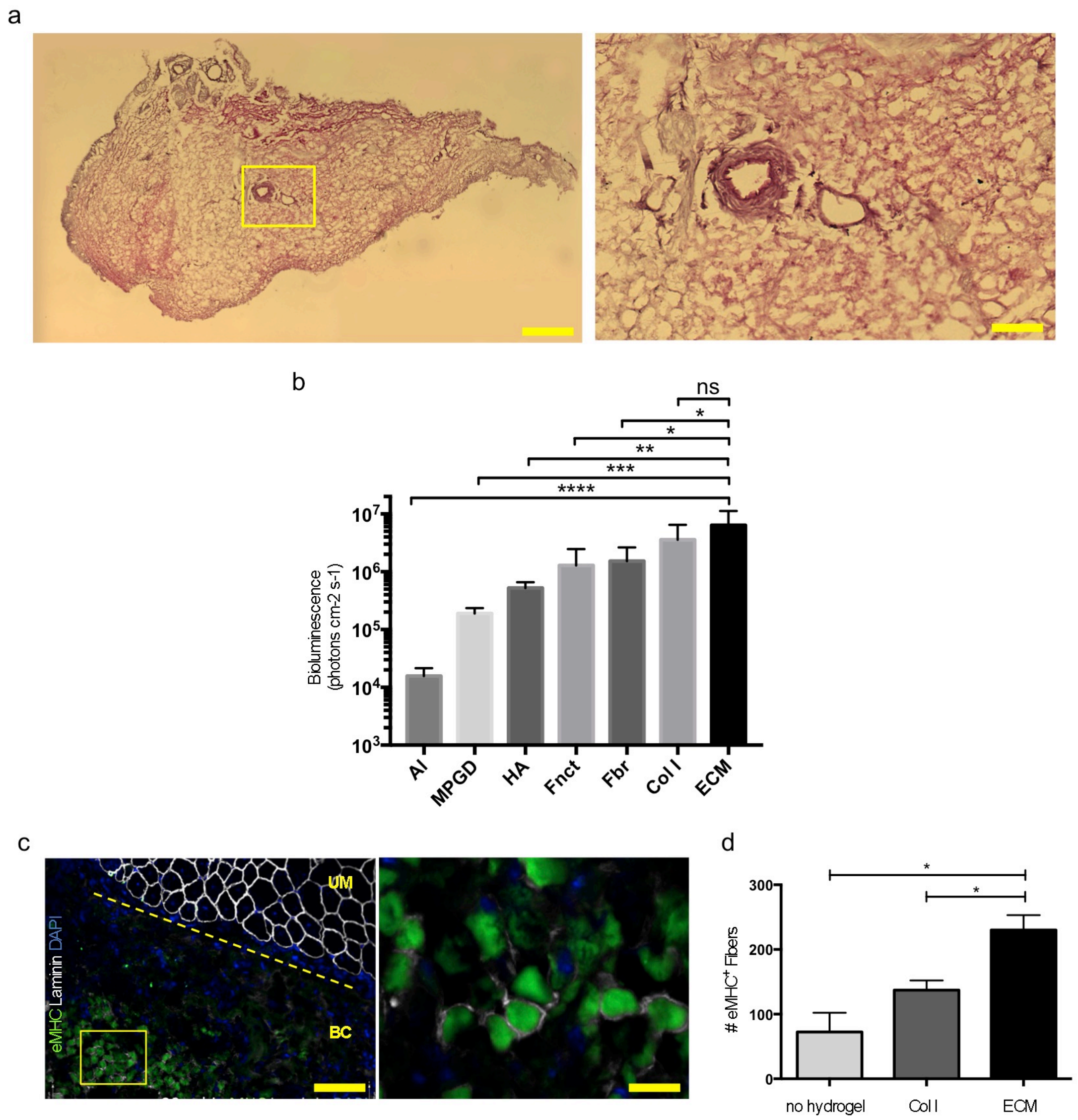

Supplementary Fig. 1.

De novo myofibers from MuSCs transplanted within ECM-scaffolds

a. Representative images of decellularized TA muscle. Whole TA muscles were chemically decellularized (left panel), resulting in acellular scaffolds (the region outlined by the yellow box is shown at higher magnification in the right panel showing the intact ECM structure remaining after decellularization). **b**. Freshly isolated MuSCs expressing Luciferase were suspended in different hydrogels and cultured. Two days later, bioluminescence was imaged. Hydrogels screened were:

Collagen I (Col I), Alginate (Al), Hyaluronic Acid (HA), Fibronectin (Fnct), methoxy-polyethylene glycol-diacylglycerol (MPGD), Fibrin (Fbr) and an ECM protein cocktail composed mostly of Collagen IV, proteoglycan, Laminin (ECM) (n = 3). **c**. Representative immunofluorescence (IF) images of *de novo* myofibers formed within transplanted scaffolds. Scaffolds were seeded with MuSCs contained within ECM-based hydrogels and then transplanted into TA muscles immediately following VML injury. After 10 days, muscles were isolated and IF was performed to stain for embryonic Myosin Heavy Chain ($eMHC^+$) fibers. A dashed line indicates the region between the transplanted bioconstruct (BC) and the remaining unablated muscle tissue (UM) (scale bar = 200 μm). The region outlined by the yellow box contains newly formed fibers, which are magnified in the right panel (left scale bar = 500 μm; right scale bar = 50 μm). **d**. Quantification of *de novo* myofibers formed within the transplanted scaffolds generated using different hydrogels. Scaffolds were reconstituted with MuSCs along with either a Collagen I (Col I) hydrogel, an ECM-based hydrogel, or alone (no hydrogel), and were then transplanted into acute VML injuries. After 10 days, muscles were isolated. IF was used to stain and quantify $eMHC^+$ fibers (n = 6). Data are ± s.e.m. For statistical analysis, t-tests were used. ** $p<0.001$; *** $p<0.0001$; **** $p<0.00001$.

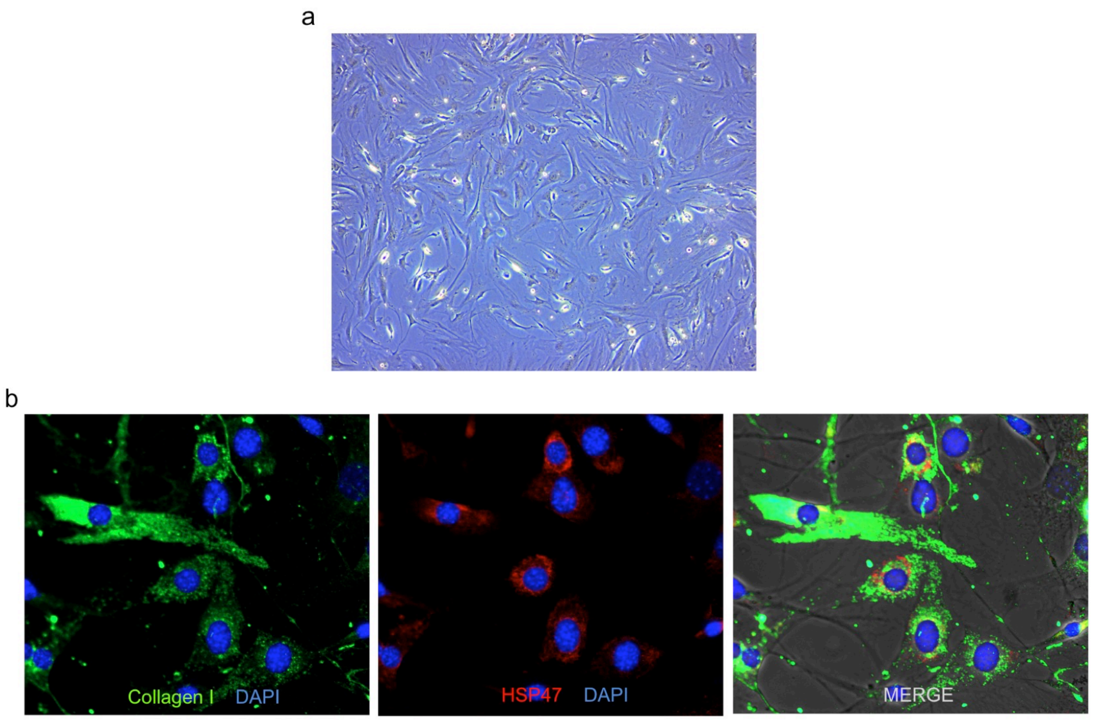

Supplementary Fig. 2.

Fibroblast-like cells are a subset of MRCs

a. Representative optical bright field image of cells cultured for 7 days that were derived from a cell population freshly isolated by FACS. These cells stained negatively for all the surface marker proteins employed in the sorting scheme ($VCAM^-/CD45^-/CD31^-/Sca1^-$). **b**. Representative immunofluorescence image of the same population as in panel (**a**), showing that these cells stained for the typical markers of fibroblast-like cells (Collagen I, HSP47).

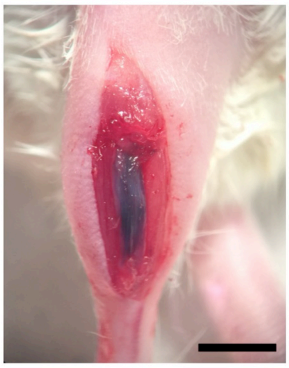

Supplementary Fig. 3.

Bioconstruct transplanted into a TA muscle with a VML injury

Photograph showing the lower limb of a mouse after transplantation of a bioconstruct containing cells and an ECM hydrogel into the TA muscle. The blue dye (Evans Blue) highlights the transplanted bioconstruct (scale bar = 10 mm)

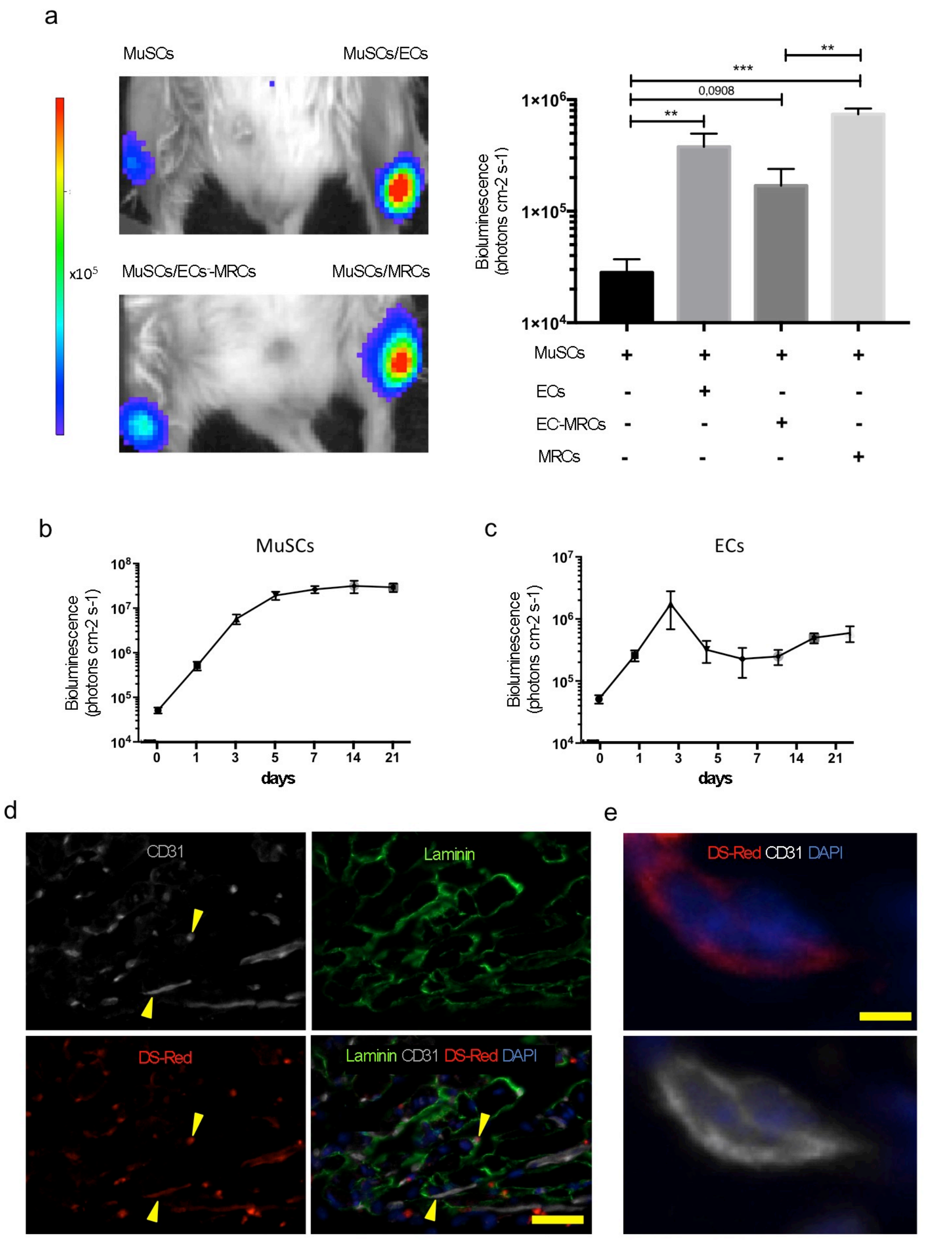

Supplementary Fig. 4.

ECs are necessary and sufficient to sustain de novo myofiber

a. Bioconstructs were generated with Luc$^+$ MuSCs alone or with unlabeled: 1) ECs alone (ECs); 2) MRCs (MRCs); or 3) MRCs depleted of ECs (EC$^-$-MRCs). These bioconstructs were transplanted into right and left TA muscles after receiving VML injuries. Left panels: representative bioluminescence images of mice 21 days after transplantation of bioconstructs into both TA muscles immediately following VML injury. Right panel: quantitative results of bioluminescence imaging of transplanted

bioconstructs (n = 3). **b**. Quantitative results comparing bioluminescence of bioconstructs generated with Luc^{+} MuSCs and Luc^{-} MRCs at periodic intervals following transplantation into VML injuries (n = 4). **c**. Quantitative results comparing bioluminescence of bioconstructs generated with Luc^{-} MuSCs and Luc^{+} ECs together with Luc^{-} EC^{-}-MRCs at periodic intervals following transplantation into VML injuries (n = 4). **d**. Representative IF images of a cross-section of a TA muscle 21 days after receiving a transplanted bioconstruct generated with $DsRED^{-}$ MuSCs and $DsRED^{+}$ ECs together with $DsRED^{-}$ EC^{-}-MRCs to treat VML injury (scale bar = 100 μm). Yellow arrowheads indicate two selected blood vessels. **e**. Higher magnification image of donor-derived ($DsRED^{+}$) vasculature structures ($CD31^{+}$) from a region of the same transplanted bioconstruct shown in panel (**d**) (scale bar = 5 μm). Data are ±s.e.m. For statistical analysis, t-tests were used. ** $p<0.001$; *** $p<0.0001$.

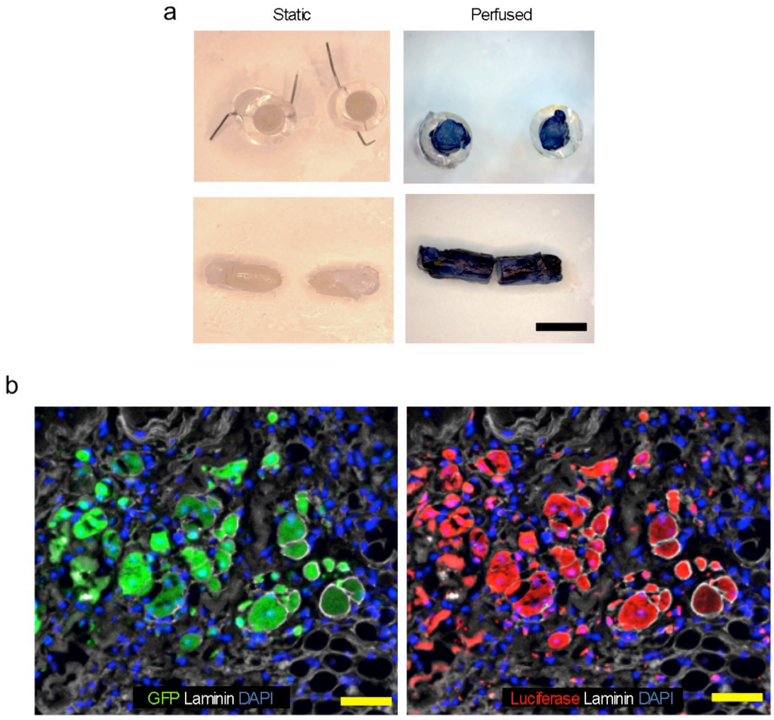

Supplementary Fig. 5.

Perfusion of bioconstructs enhances de novo fiber formation

a. Representative images of bioconstructs before and after perfusion. Bioconstructs were placed within tubular culture chambers and connected to a bioreactor (Fig. 3b), which allowed media to perfuse the bioconstructs. The images on the left are those of a bioconstruct under static conditions seen both in cross-section, located within a tubular culture chamber (top panel), and longitudinally, after being removed from the culture chamber (bottom panel). The images on the right are those of a bioconstruct that has been perfused by media containing a blue dye (Evans Blue) (scale bar = 5 mm). **b.** Perfused bioconstructs generated with GFP^{+}/Luc^{+} MuSCs were transplanted into VML-injured TA muscles and analyzed for GFP and Luciferase expression 10 days later (scale bar = 100 μm).

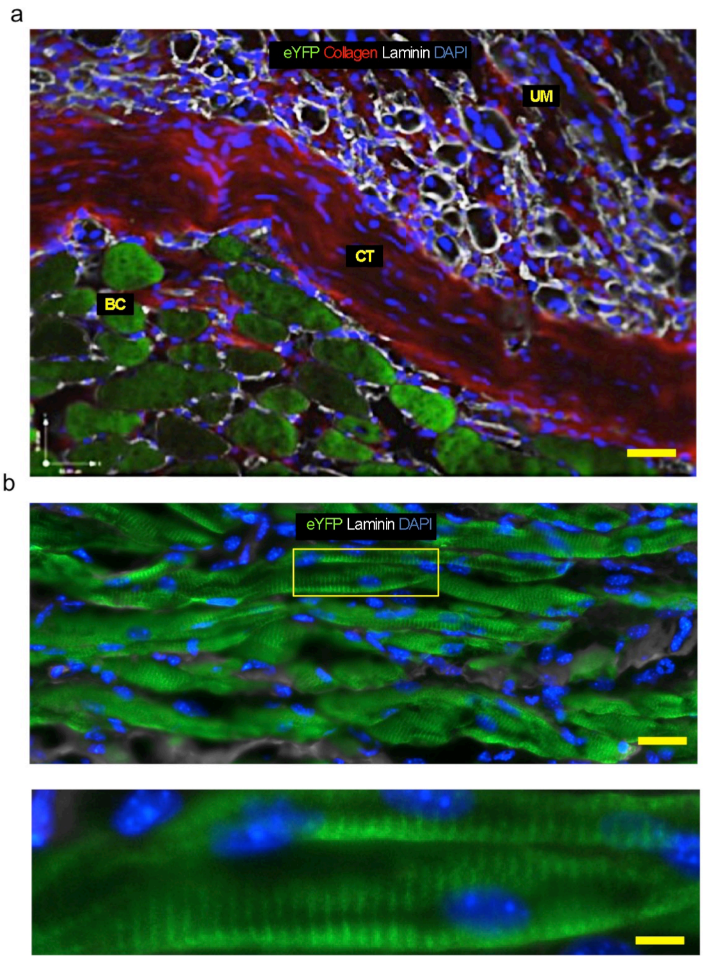

Supplementary Fig. 6.

Interface between de novo donor-derived tissue and host muscle

a. Representative image of a region of a transplanted bioconstructs within a VML lesion and adjacent to normal muscle. This particular bioconstruct was generated using eYFP$^+$ MuSCs and eYFP$^-$ MRCs. Imaging was performed 10 days after transplantation. Donor-derived myofibers are eYFP$^+$ and located within the region of the transplanted bioconstruct. Also within the field of view is unablated muscle (UM) tissue, separated from the bioconstructs (BC) by connective tissue (CT) (scale bar = 100 µm). **b**. Representative IF longitudinal images showing donor-derived (eYFP$^+$) myofibers in VML-injured TA muscles following treatment (scale bar = 500 µm). The yellow box magnified in the lower panel shows striated donor-derived (eYFP$^+$) myofibers (scale bar = 5 µm).

a

b

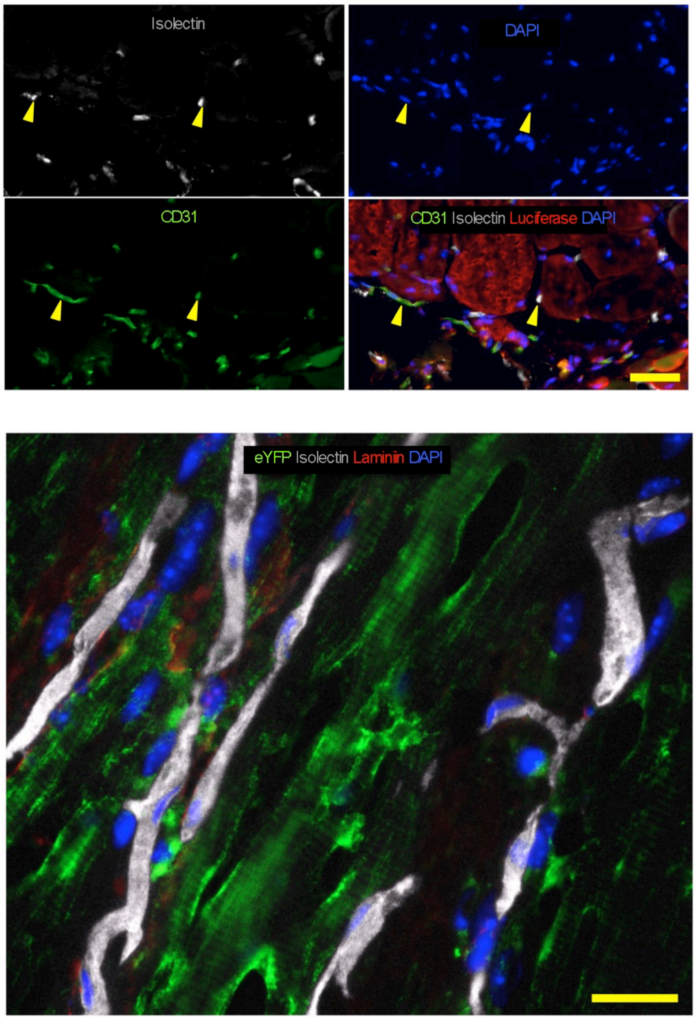

Supplementary Fig. 7.

De novo vasculature is functionally engrafted with the host

a. Representative cross-sectional images of *de novo* tissue within transplanted bioconstructs. Mice received Luc$^+$ MuSC/Luc$^-$ MRC bioconstructs transplanted in TA muscles with VML injuries and were injected intravenously via the lateral tail vein 30 days later with biotinylated-Isolectin. TA muscles were fixed 10 minutes after injection and were then analyzed by IF staining. Yellow arrowheads indicate two selected blood vessels (CD31$^+$) staining positive for Isolectin and adjacent to donor-derived (Luc$^+$) myofibers (scale bar = 100 μm). **b**. Representative longitudinal section image of *de novo* tissue within transplanted eYFP$^+$ MuSC/ eYFP$^-$ MRC bioconstructs (scale bar = 100 μm).

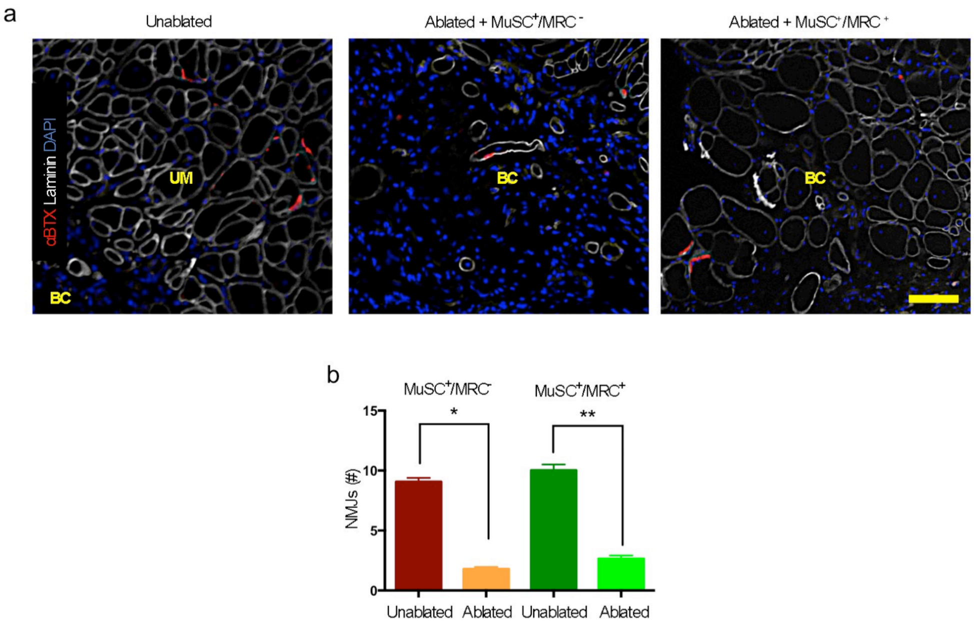

Supplementary Fig. 8.

Innervation of de novo myofibers within transplanted bioconstructs

a. Representative IF images of neuromuscular junctions (NMJs) within a transplanted bioconstruct following VML injury. After 30 days, muscles were stained for the presence of NMJs using fluorescently-labeled αBTX. The number of NMJs within the regions of transplanted bioconstructs (BC) were compared to regions of unablated muscle (UM) (scale bar = 100 μm). **b.** Quantification of mature NMJs within treated ablated regions compared to unablated regions of the same muscles (n = 6). Data are ±s.e.m. For statistical analysis, t-tests were used. * $p<0.05$; ** $p<0.001$.

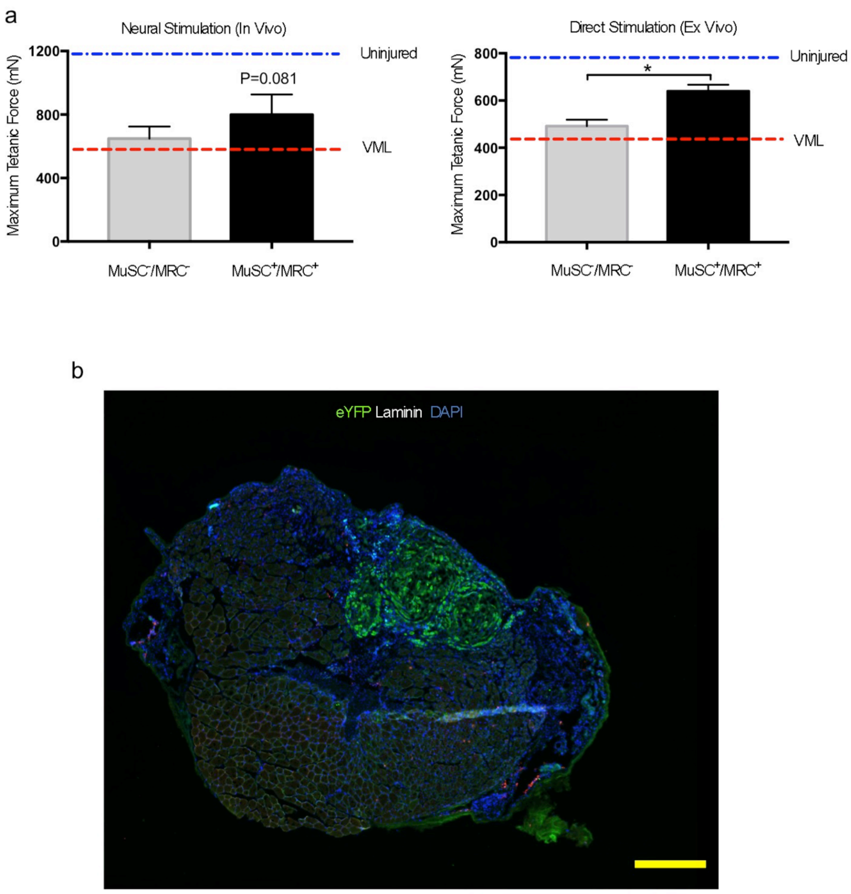

Supplementary Fig. 9.

Force production two months after treatment of VML injuries

a. VML injuries were induced in TA muscles which were then transplanted with bioconstructs containing no cells (MuSC$^-$/MRC$^-$) or containing eYFP$^+$ MuSCs and eYFP$^-$ MRCs (MuSC$^+$/MRC$^+$). After 60 days, force production was measured *in vivo* (left graph) and *ex vivo* (right graph) (n = 3). **b**. Representative IF images of cross-sections of VML-injured TA muscles treated with bioconstructs generated with eYFP$^+$ MuSCs and eYFP$^-$ MRCs and analyzed for force productions in panel (**a**) (scale bar = 1 mm). Data are ±s.e.m. For statistical analysis, t-tests were used. * $p<0.05$; ** $p<0.001$.

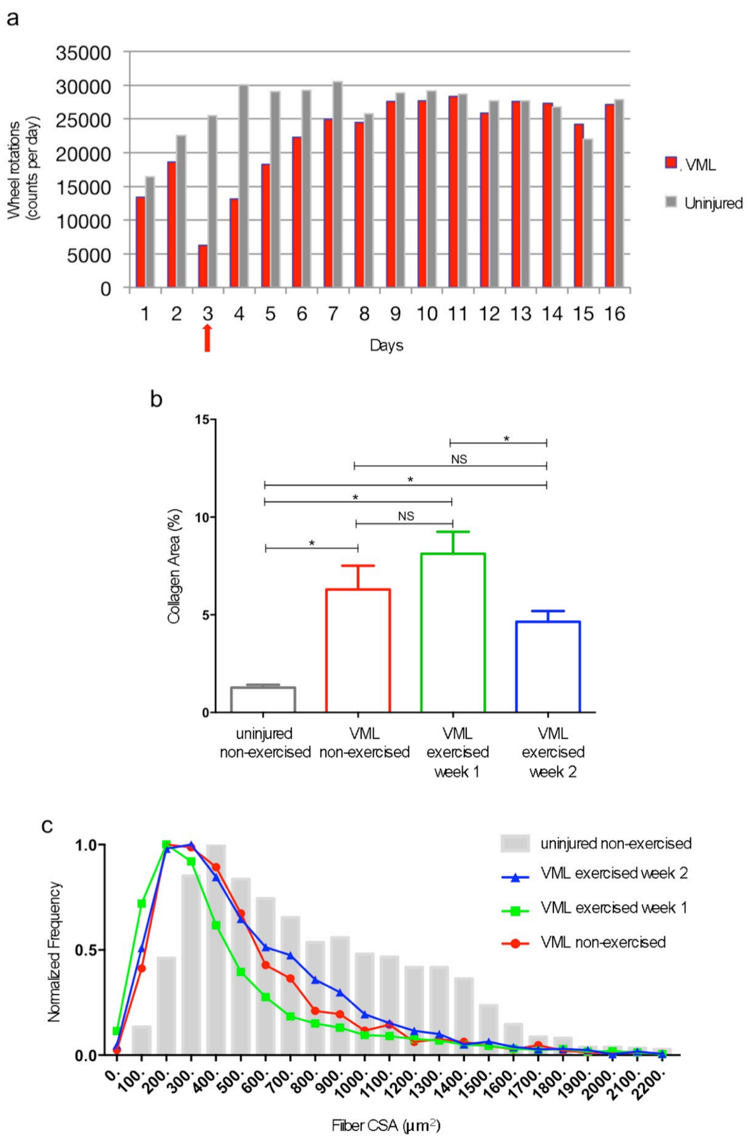

Supplementary Fig. 10.

Exercise improves function and structure in VML

a. Time course of daily voluntary running activity in mice with TA muscles that received VML injuries at day 3 (red arrow) or in uninjured controls. **b**. Mice received VML injuries to both TA muscles and were challenged to run for one week either immediately after injury or after one week of rest and were compared to injured or uninjured non-exercised mice. At 14 days after the injuries, muscles were fixed, immunostained for Collagen I and Laminin and analyzed for fibrotic areas (Collagen^{+}) in whole TA CSAs (n = 5). **c**. Morphometric analysis of myofiber CSAs of the same TA muscles analyzed in panel (**b**). Data are ±s.e.m. For statistical analysis, t-tests were used. * $p<0.05$.

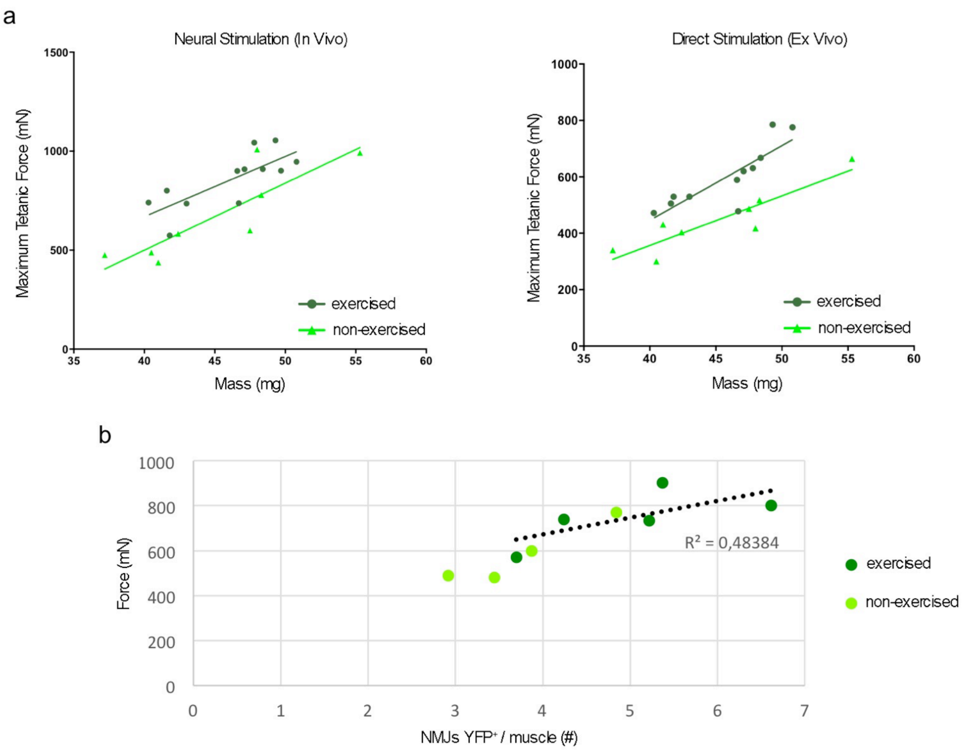

Supplementary Fig. 11.

Exercise improves function and innervation in treated VML injuries

a. Correlation between masses and *in vivo* forces (left) or *ex vivo* forces (right) of individual muscles that received VML injuries and treatment with bioconstructs. Measurements were conducted 30 days following treatment in exercised and non-exercised mice (in the graphs, each dot corresponds to a mouse). **b**. Correlation between the average numbers of NMJs associated with *de novo* myofibers and force production *in vivo* for individual muscles. Measurements and analyses were conducted 30 days following treatment in exercised and non-exercised mice (in the graph, each dot corresponds to a mouse).

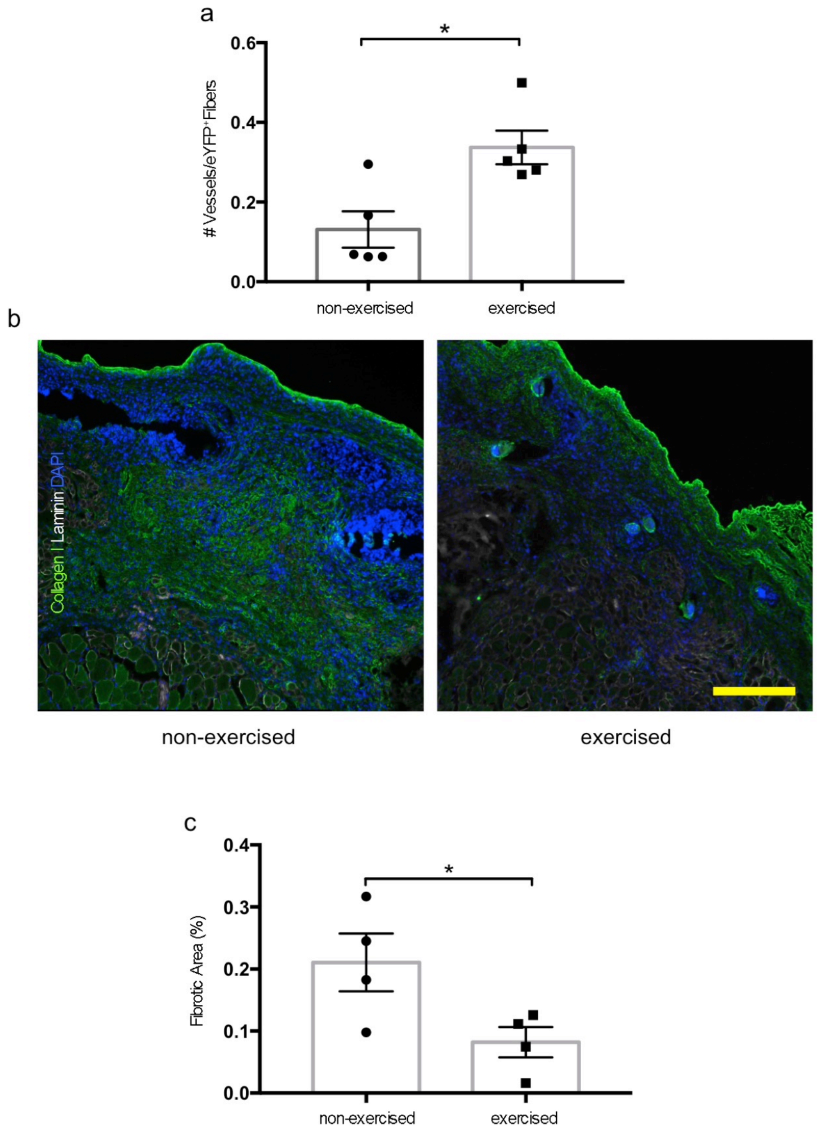

Supplementary Fig. 12.

Exercise increases vasculogenesis and reduces fibrosis

a. Mice that received VML injuries to TA muscles and that were transplanted with bioconstructs containing both MuSCs and MRCs were either exercised for three weeks after a week of rest or not exercised. After 30 days, muscles were harvested for IF analysis. Within transplanted bioconstruct regions with donor-derived myofibers, the numbers of vasculature structures were quantified. Data are ±s.e.m. For statistical analysis, t-tests were used. * $p<0.05$ (in the graph, each dot corresponds to a mouse).

b. Representative IF images of cross-sections of VML-injured TA muscle regions

within transplanted MuSC/MRC bioconstructs from exercised or non-exercised mice. Mice were either exercised for three weeks after a week of rest or not exercised. After 30 days, muscles were immunostained for Collagen I (scale bar = 500 μm). **c**. Quantification of fibrotic areas (Collagen I^{+}) in whole regions of transplanted bioconstructs of muscles with treated VML injures, as described in (**b**). Data are ±s.e.m. For statistical analysis, t-tests were used. * $p<0.05$ (in the graph, each dot corresponds to a mouse).

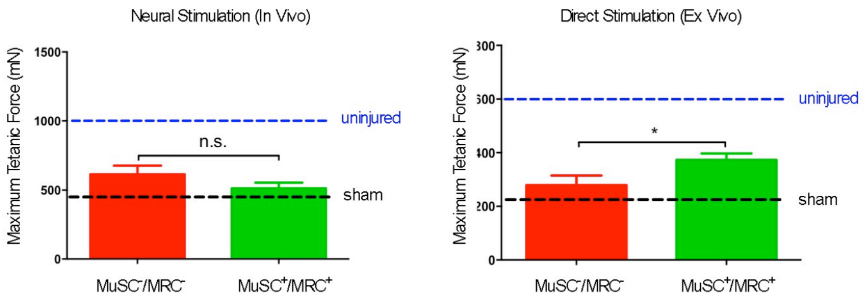

Supplementary Fig. 13.

Bioconstructs enhance function in muscle with VML chronic injuries

VML injury was induced in TA muscles and then allowed to heal for 30 days, at which point the injured muscle was transplanted with bioconstructs containing both MuSCs and MRCs. After an additional 30 days, force production was measured *in vivo* and *ex vivo* (n=6). Data are ±s.e.m. For statistical analysis, t-tests were used. * $p<0.05$.

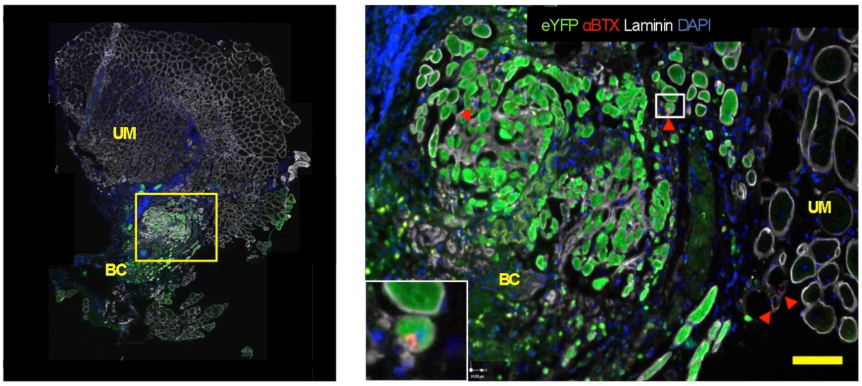

Supplementary Fig. 14.

Muscle structure after bioconstruct treatment of chronic VML injuries

(Left) Representative images of a TA muscle with a chronic VML injury treated with a transplanted bioconstruct. One of the muscles that was fixed, stained, imaged and analyzed in Supplementary Fig. 14 is shown. Bioconstructs were generated with $eYFP^+$ MuSCs and $eYFP^-$ MRCs. The left panel shows a cross-section of a whole TA muscle including unablated muscle (UM) after chronic VML injury and treatment with a bioconstruct (BC). (Right) Higher magnification of the region designated by the yellow box in the left panel. Red arrowheads indicate all the NMJs (αBTX^+) associated with donor-derived myofibers within the area. Inset shows a higher magnification of a donor-derived ($eYFP^+$) myofiber with a NMJ (αBTX^+) outlined by the white box (scale bar = 50 μm).

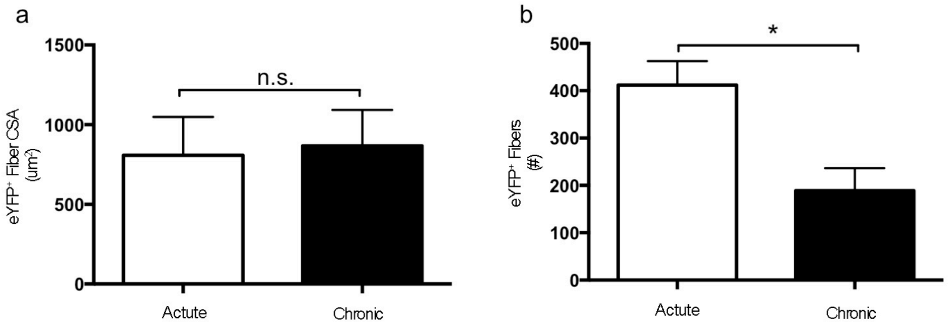

Supplementary Fig. 15.

Comparison between treated acute and chronic VML injuries

a. Quantification of CSAs of eYFP$^+$ myofibers generated from transplanted bioconstructs in TA muscles with acute or chronic VML injuries (n = 6). **b**. Quantification of the total number of eYFP$^+$ myofibers analyzed as in panel (**a**) (n = 6). Data are ±s.e.m. For statistical analysis, t-tests were used. * $p<0.05$.

Compound	Concentration	Product ID	Reference
Collagen	2.5 mg/ml	Sigma, #C8919	Quarta et al., *Nature Biotechnol.*, 2016 [1]
Alginate	3 g/ml	NovaMatrix, #4202006	Lee and Mooney, *Prog. Polym. Sci.*, 2012 [2]
Hyaluronic Acid	1 mg/ml	Advanced Biomatrix, #GS313-1EA	According to the manufacturer
Fibronectin	3 mg/ml	MAPTrix, #161101	According to the manufacturer
Methoxy-polyethyleneglycol-diacylglycerol	1 mg/ml	MAPTrix HyGel, #361051	According to the manufacturer
HyStem-C	Non-specified by the manufacturer	ESI-BIO, #GS313	According to the manufacturer
Fibrinogen	8 mg/ml	Sigma-Aldrich, #F3879	Shachaf et al., *Biomaterials*, 2012 [3]
Thrombin	1:10 ratio with Fibrinogen	Sigma-Aldrich, #T6884	Duong et al., *Tissue Eng. Part*, 2009 [4]
Muscle-derived ECM	1 mg/ml	Made in the lab	Ungerleider et al., *Methods*, 2015 [5]

Table 1. **Hydrogel compositions used to generate cell-encapsulating matrices**.

References

1. Quarta, M. *et al.* An artificial niche preserves the quiescence of muscle stem cells and enhances their therapeutic efficacy. *Nat. Biotechnol.* **34,** 752–759 (2016).
2. Lee, K. Y. & Mooney, D. J. Alginate: properties and biomedical applications. *Prog Polym Sci* **37,** 106–126 (2012).
3. Shachaf, Y., Gonen-Wadmany, M. & Seliktar, D. The biocompatibility of PluronicF127 fibrinogen-based hydrogels. *Biomaterials* **31,** 2836–2847 (2010).
4. Duong, H., Wu, B. & Tawil, B. Modulation of 3D fibrin matrix stiffness by intrinsic fibrinogen-thrombin compositions and by extrinsic cellular activity. *Tissue Eng Part A* **15,** 1865–1876 (2009).
5. Ungerleider, J. L., Johnson, T. D., Rao, N. & Christman, K. L. Fabrication and characterization of injectable hydrogels derived from decellularized skeletal and cardiac muscle. *Methods* **84,** 53–59 (2015).
